# Screening and diagnosis of cardiovascular disease using artificial intelligence-enabled cardiac magnetic resonance imaging

**DOI:** 10.1038/s41591-024-02971-2

**Published:** 2024-05-13

**Authors:** Yan-Ran (Joyce) Wang, Kai Yang, Yi Wen, Pengcheng Wang, Yuepeng Hu, Yongfan Lai, Yufeng Wang, Kankan Zhao, Siyi Tang, Angela Zhang, Huayi Zhan, Minjie Lu, Xiuyu Chen, Shujuan Yang, Zhixiang Dong, Yining Wang, Hui Liu, Lei Zhao, Lu Huang, Yunling Li, Lianming Wu, Zixian Chen, Yi Luo, Dongbo Liu, Pengbo Zhao, Keldon Lin, Joseph C. Wu, Shihua Zhao

**Affiliations:** 1 School of Medicine, Stanford University, Stanford, CA USA; 2 Department of Magnetic Resonance Imaging, Fuwai Hospital and National Center for Cardiovascular Diseases, Chinese Academy of Medical Sciences and Peking Union Medical College, Beijing, China; 3 Changhong AI Research (CHAIR), Sichuan Changhong Electronics Holding Group, Mianyang, China; 4 Department of Biomedical Engineering, University of Southern California, Los Angeles, CA USA; 5 Department of Electrical and Computer Engineering, Duke University, Durham, NC USA; 6 School of Engineering, University of Science and Technology of China, Hefei, China; 7 Department of Computer Science, Stony Brook University, New York, NY USA; 8 Paul C. Lauterbur Research Center for Biomedical Imaging, Shenzhen Institutes of Advanced Technology, Chinese Academy of Sciences, Shenzhen, China; 9 Department of Electrical Engineering, Stanford University, Stanford, CA USA; 10 Stanford Cardiovascular Institute, School of Medicine (Division of Cardiology), Stanford University, Stanford, CA USA; 11 Peking Union Medical College Hospital, Beijing, China; 12 Guangdong Provincial People’s Hospital, Guangzhou, China; 13 Beijing Anzhen Hospital, Beijing, China; 14 Tongji Hospital, Wuhan, China; 15 The Second Affiliated Hospital of Harbin Medical University, Harbin, China; 16 Renji Hospital, Shanghai, China; 17 The First Hospital of Lanzhou University, Lanzhou, China; 18 The First Affiliated Hospital of USTC, Division of Life Sciences and Medicine, University of Science and Technology of China, Hefei, China; 19 Department of Electrical and Computer Engineering, Northwestern University, Evanston, IL USA; 20 Mayo Clinic Alix School of Medicine, Phoenix, AZ USA

**Keywords:** Cardiovascular diseases, Translational research, Image processing, Magnetic resonance imaging, Machine learning

## Abstract

Cardiac magnetic resonance imaging (CMR) is the gold standard for cardiac function assessment and plays a crucial role in diagnosing cardiovascular disease (CVD). However, its widespread application has been limited by the heavy resource burden of CMR interpretation. Here, to address this challenge, we developed and validated computerized CMR interpretation for screening and diagnosis of 11 types of CVD in 9,719 patients. We propose a two-stage paradigm consisting of noninvasive cine-based CVD screening followed by cine and late gadolinium enhancement-based diagnosis. The screening and diagnostic models achieved high performance (area under the curve of 0.988 ± 0.3% and 0.991 ± 0.0%, respectively) in both internal and external datasets. Furthermore, the diagnostic model outperformed cardiologists in diagnosing pulmonary arterial hypertension, demonstrating the ability of artificial intelligence-enabled CMR to detect previously unidentified CMR features. This proof-of-concept study holds the potential to substantially advance the efficiency and scalability of CMR interpretation, thereby improving CVD screening and diagnosis.

## Abstract (web summary)

A two-step, video-based deep learning model is developed to first screen for cardiac anomalies using noncontrast magnetic resonance imaging, followed by diagnosis of 11 types of cardiovascular disease using gadolinium enhancement-based imaging.

## Main

Cardiovascular diseases (CVDs) are the number one leading cause of death in the world^1^. According to the World Health Organization, an estimated 17.9 million people die each year from CVDs, accounting for approximately 32% of all deaths worldwide. Among these, over 75% of CVD deaths occur in low- and middle-income countries^2,3^. Although multiple approaches can be used to diagnose CVDs, cardiac magnetic resonance imaging (CMR) is a comprehensive imaging modality well suited to evaluate cardiac morphology, function, myocardial perfusion and unique tissue characterization^4–7^. As a result, CMR is considered the gold standard for assessing cardiac function and diagnosing CVDs^8–11^. However, widespread clinical implementation of CMR has been hindered by the time cost of CMR interpretation, considerable training time and efforts to gain the expertise, and the resulting shortage of qualified CMR-trained doctors^12^. The limited availability of adequately trained CMR experts can make timely and accurate diagnosis of CVDs using CMR extremely difficult. Consequently, the use of automated CMR interpretation for the rapid screening and diagnosis of CVDs demonstrates great clinical potential^13^.

The ability of deep learning to learn distinctive features and recognize motion patterns from raw input images and videos without requiring hand-crafted feature engineering^14^ and extensive data preprocessing makes it highly effective for interpreting CMR data. Furthermore, deep learning algorithms have a clear advantage over humans by analyzing all images and dynamic pieces of information simultaneously and uniformly^15^, offering more efficient and objective solutions. However, a comprehensive evaluation of whether an end-to-end deep learning approach can be used to analyze CMR data to screen for and diagnose a broad range of CVDs remains lacking^16^. The few applications of deep learning in CMR so far have focused on single aspects of CMR interpretation (for example, segmentation^17–19^ or wall thickness measurement^20^) or have demonstrated limited diagnostic capabilities (for example, myocardial scarring or aortic valve malformations^21–23^).

**Fig. 1:**
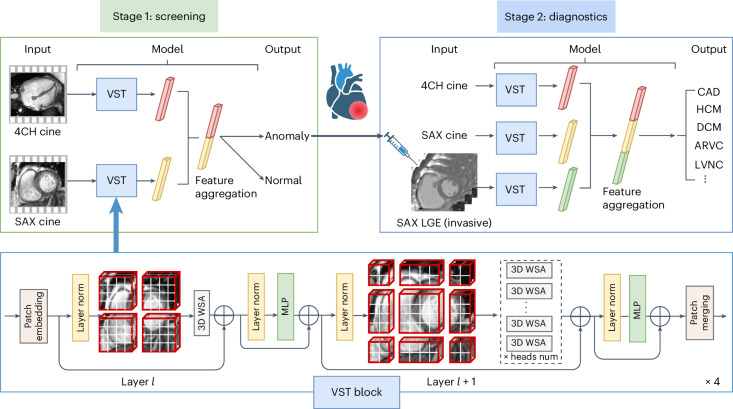
Workflow of the two-stage paradigm for automatic screening and diagnostics of CVDs. For each patient, the screening model takes cine movies as inputs and outputs the binary classification to detect cardiac anomaly. Second, the patient suspected of cardiac anomaly undergoes LGE imaging, while the diagnostic model integrates both cine and LGE to output their CVD class. The AI models comprised four VST blocks to analyze the CMR sequences using the 3D-shifted window self-attention (WSA) mechanism. MLP, multilayer perceptron; norm, normalization; num, number; layer *l* and layer *l* + 1 indicate two consecutive layers.

In this Article, we aimed to develop and validate a deep learning approach for automatic, computerized CMR interpretation and diagnosis consisting of a two-stage paradigm that mimics the clinical workflow: (1) screening for anomalies using nonenhanced cine magnetic resonance imaging (MRI) followed by (2) diagnosing CVDs using cine and late gadolinium enhancement (LGE) MRI as combined inputs. The initial stage, based on cine modality, enables a noninvasive cardiac screening. Compared with LGE, which requires the injection of a gadolinium contrast agent^24^, cine MRI is safer and more easily acquired. The second stage provides classification of 11 types of CVDs covering most patients referred to the CMR examination^25^ (ischemic heart disease, most types of nonischemic cardiomyopathy^26^, pulmonary hypertension and congenital heart disease; Table 1). We propose video-based swin transformer (VST)^27^—a cutting-edge advancement in computer vision—as our model backbone of choice instead of the conventional convolutional neural network (CNN) approach, and highlighted the superiority of the transformer model in modelizing CMR sequences. The proposed automatic pipeline consists of two serial VST-based artificial intelligence (AI) models: the screening model and the diagnostic model (Fig. 1). Further, we examined which imaging modality (cine or LGE), view (four chamber or short axis) and their aggregation should be utilized for optimal classification performance. Finally, we compared the performance of the AI model with physicians of varying experience in CMR interpretation. This study creates an avenue for accurate CMR interpretation in real time, as well as bringing CMR into more widespread use in CVD screening and diagnosis.

**Table 1:** Characteristics of the primary and external test datasets

		Primary dataset				External test dataset				Entire dataset
		No. of subjects	Sex		Age in years (range)	No. of subjects	Sex		Age in years (range)	
			Male	Female			Male	Female		
Total		7,900	5,380 (68%)	2,520 (32%)	45 ± 16 (2–86)	1,819	1,228 (68%)	591 (32%)	47 ± 16 (1–88)	9,719
Normal control cohort		1,250	700 (56%)	550 (44%)	37 ± 14 (10–78)	403	230 (57%)	173 (43%)	41 ± 16 (6–79)	1,653
CVD cohort		6,650	4,680 (70%)	1,970 (30%)	47 ± 15 (2–86)	1,416	998 (71%)	418 (29%)	48 ± 16 (1–88)	8,066
1	HCM	2,327	1,513 (65%)	814 (35%)	48 ± 14 (7–86)	388	260 (67%)	128 (33%)	51 ± 15 (9–86)	2,715
2	DCM	1,435	1,076 (75%)	359 (25%)	44 ± 15 (4–82)	204	140 (69%)	64 (31%)	50 ± 14 (8–76)	1,639
3	CAD	942	829 (88%)	113 (12%)	56 ± 11 (8–83)	299	269 (90%)	30 (10%)	56 ± 11 (24–88)	1,241
4	LVNC	291	192 (66%)	99 (34%)	39 ± 16 (6–77)	30	18 (60%)	12 (40%)	40 ± 14 (11–65)	321
5	RCM	355	170 (48%)	185 (52%)	50 ± 20 (7–85)	22	13 (59%)	9 (41%)	38 ± 24 (1–78)	377
6	CAM	220	156 (71%)	64 (29%)	56 ± 11 (18–83)	138	92 (67%)	46 (33%)	59 ± 9 (29–82)	358
7	HHD	402	366 (91%)	36 (9%)	42 ± 13 (12–75)	107	88 (82%)	19 (18%)	45 ± 14 (21–75)	509
8	Myocarditis	87	64 (74%)	23 (26%)	28 ± 11 (14–69)	66	48 (73%)	18 (27%)	26 ± 12 (8–68)	153
9	ARVC	370	245 (66%)	125 (34%)	39 ± 14 (9–74)	54	37 (68%)	17 (32%)	40 ± 14 (13–67)	424
10	PAH	134	36 (27%)	98 (73%)	32 ± 12 (10–72)	66	22 (33%)	44 (67%)	38 ± 17 (10–72)	200
11	Ebstein’s anomaly	87	33 (38%)	54 (62%)	34 ± 16 (2–63)	42	11 (26%)	31 (74%)	32 ± 14 (6–61)	129

## Results

### Datasets and study design

**Extended Data Fig. 1:**
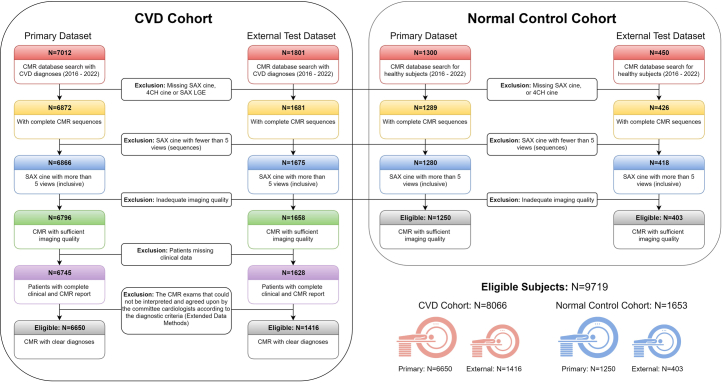
Inclusion-exclusion cascade for the cardiovascular disease and the normal control cohorts. CVD, cardiovascular disease; CMR, cardiovascular magnetic resonance imaging; SAX, short-axis; 4CH, four-chamber; LGE, late gadolinium enhancement.

We curated a nationwide, large representative CMR dataset of 9,719 individuals (6,608 male and 3,111 female) from eight medical centers across China. The dataset was divided into the CVD cohort and the normal control cohort. The disease cohort comprised 8,066 patients with CVD (mean (±s.d.) age 47.2 ± 15 years, 70% male, admitted between 2016 and 2022). Eleven types of CVDs were incorporated with the following distribution: hypertrophic cardiomyopathy (HCM; 2,715), dilated cardiomyopathy (DCM; 1,639), coronary artery disease (CAD; 1,241), left ventricular noncompaction cardiomyopathy (LVNC; 321), restrictive cardiomyopathy (RCM; 377), cardiac amyloidosis (CAM; 358), hypertensive heart disease (HHD; 509), myocarditis (153), arrhythmogenic right ventricular cardiomyopathy (ARVC; 424), pulmonary arterial hypertension (PAH; 200) and Ebstein’s anomaly (129). The baseline CMR scan (pretreatment) of each patient, with short-axis (SAX) cine, four-chamber (4CH) cine and SAX LGE all available, was collected to establish the disease cohort. In addition, the SAX cine and 4CH cine of 1,653 normal subjects (age 38 ± 15 years, 56% male, enrolled between 2016 and 2022) were collected to assemble the normal control cohort without CVDs, allowing us to develop and validate the noninvasive screening model. Table 1 and Extended Data Table 1 contain the summary statistics and the demographics of the datasets. The inclusion–exclusion cascade is summarized in Methods and Extended Data Fig. 1.

**Extended Data Fig. 2:**
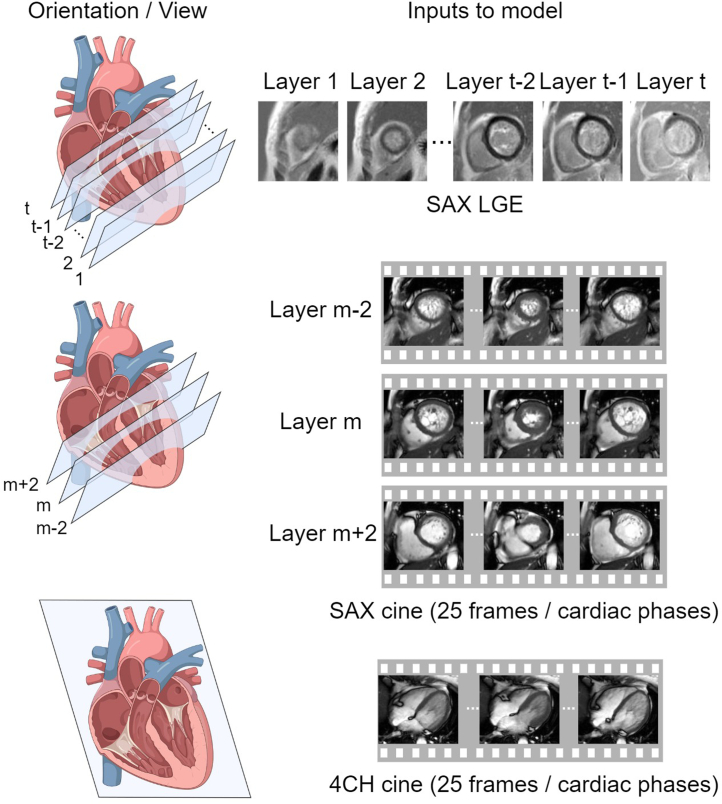
The demonstration of cardiac MRI used in developing screening and diagnostic models. The left column shows the orientation/views of the heart from which the CMR inputs are acquired. The right column lists the input images of each modality. SAX LGE is a 3D static volume, and cine is a video sequence of 25 frames. CMR, cardiovascular magnetic resonance imaging; SAX, short-axis; LGE, late gadolinium enhancement.

For the data acquisition, cardiac MRI was performed using three vendors with the following distribution: GE Healthcare (4,569), Philips (3,683) and Siemens (1,467). Cine sequence was performed in SAX orientation covering the whole left ventricle (LV) (SAX cine), as well as in long-axis covering the two-chamber, three-chamber and 4CH view. All cine sequences were 25 frames (cardiac cycle). LGE images cover the LV from the apex to the base (SAX LGE). We report performance as assessed from two major views of cine examination: SAX cine and 4CH cine, as well as SAX LGE (Extended Data Fig. 2). Supplementary Videos 1–11 show video and image examples for each class.

We used the CMR data from the Beijing Fuwai Hospital^28^ as the primary dataset for model development and data pooled from all the other medical centers as external test sets. For both screening and diagnostics, threefold cross-validation was performed within the primary dataset to further validate performance. This involved a total of 7,900 subjects and 6,650 CVD patients from the primary dataset contributing to the training of the screening and diagnostic models, respectively. Each fold of cross-validation employed 5,267 patients for screening model training and 4,433 for diagnostic model training. Overall, the screening and diagnostic models were tested with 9,719 and 8,066 patients (internal and external), respectively, and included patients from eight medical centers and CMR acquired from three different MRI vendors.

### Evaluation of screening model

**Fig. 2:**
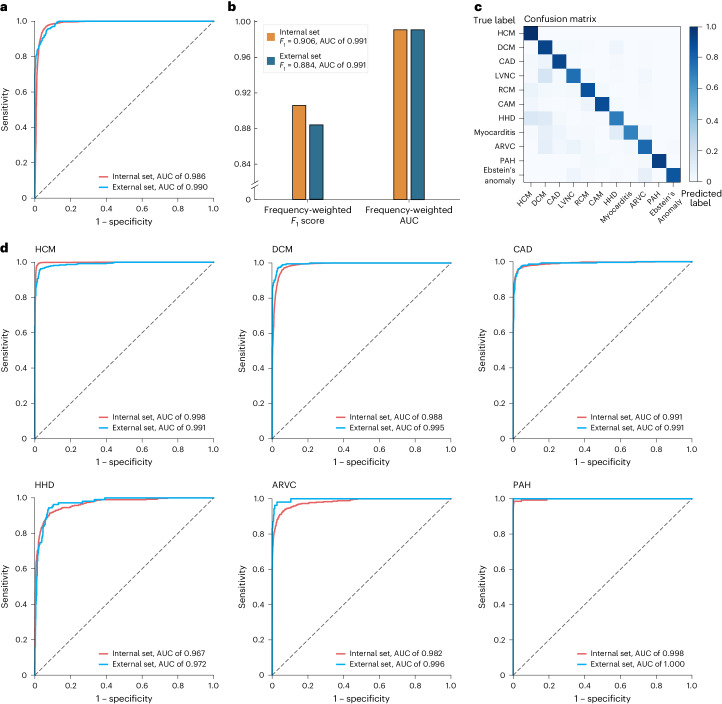
Performance of the screening and diagnostic models in internal and external testing. **a**, ROCs for the screening of cardiac anomalies for the primary internal test dataset (blue, *n* = 7,900) and external test dataset (red, *n* = 1,819). The screening model is derived from 4CH cine and SAX cine. **b**, The diagnostic performance for the internal test dataset (yellow, *n* = 6,650) and external test dataset (blue, *n* = 1,416). The diagnostic model takes cine (4CH and SAX) and LGE as combined inputs. **c**, A confusion matrix for the predictions of the AI diagnostic model versus the ground truth over the entire CVD cohort (*n* = 8,066). The percentage of all possible predictions in each CVD class is displayed on a color gradient scale. **d**, ROCs for the diagnosis of CVD classes for the internal set and external set.

The screening model with cine MRI from two combined views (SAX cine and 4CH cine) achieved an area under the curve (AUC) of 0.986 (95% confidence interval (CI) 0.984–0.988) and *F*_1_ score of 0.977 (95% CI 0.974–0.979) for screening on the threefold cross-validation upon the primary dataset (*n* = 7,900) (Fig. 2 and Extended Data Table 2). The sensitivity of 0.973 (95% CI 0.968–0.978) was achieved by the model for anomaly detection with specificity at 90%. All sensitivity and specificity pairs were >90%. It is worth noting that the primary dataset contained a wide spectrum of CVDs (11 types; Table 1), demonstrating the robustness of the screening model with respect to disease type.

In the evaluation of each view of cine for screening, the model derived from 4CH view received an AUC of 0.974 (95% CI 0.969–0.979) and the model derived from SAX view received an AUC of 0.971 (95% CI 0.965–0.976). The combination of SAX and 4CH cine together provided the best performance in comparison to models derived from single-view input (Extended Data Table 2). Note that greater than 95% sensitivity was achieved by both single-view models for anomaly detection with specificity at 90% (Extended Data Table 2). This demonstrates the potential of fast screening based on cine sequence from either SAX or 4CH view.

### Evaluation of diagnostic model

Next, we developed the diagnostic model to classify 11 CVD classes. Cine from both views (SAX and 4CH cine) and SAX LGE are combined inputs to the diagnostic model to ensure that any piece of complementary information present in CMR is effectively used to improve the diagnostic accuracy. Upon threefold cross-validation in the primary dataset (*n* = 6,650), the model achieved a class-weighted average AUC of 0.991 and *F*_1_ score of 0.906 (Fig. 2 and Extended Data Table 3). The model achieved an AUC of greater than 0.96 for all classes; for all classes, all but three (LVNC, HHD and myocarditis) had *F*_1_ scores above 0.80. The model demonstrated high AUCs and *F*_1_ scores for the most prevalent CVDs including HCM (AUC 0.998, 95% CI 0.997–0.999; *F*_1_ 0.975, 95% CI 0.971–0.980), DCM (AUC 0.988, 95% CI 0.986–0.990; *F*_1_ 0.896, 95% CI 0.884–0.907) and CAD (AUC 0.991, 95% CI 0.988–0.994; *F*_1_ 0.921, 95% CI 0.908–0.935). The PAH class also had a high AUC of 0.998 (95% CI 0.995–1.000) and *F*_1_ score of 0.962 (95% CI 0.937–0.984).

**Fig. 3:**
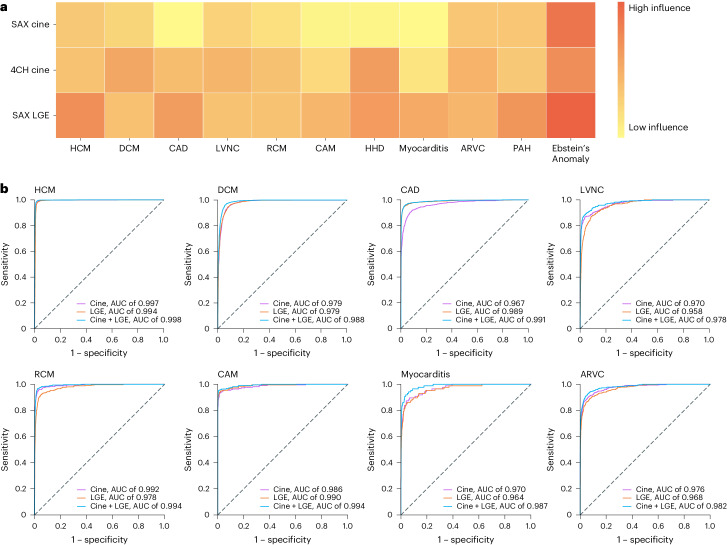
Influences of individual CMR modalities. **a**, Shapley values of SAX cine, 4CH cine and SAX LGE, derived from the diagnostic model (cine and LGE as combined inputs) for the prediction of each CVD class. Shapley values are displayed on a color gradient scale, with red indicating the CMR modality with the greatest influence for each CVD classification. The CMR modalities, exhibiting characteristic features for the diagnosis of the CVD class, demonstrate a consistently strong impact on their model prediction: SAX LGE for the diagnosis of CAD (distinct feature: the endomyocardial or transmural LGE matching the area of coronary artery dominance); SAX LGE for HCM (hypertrophy and RV insertion point LGE); SAX LGE for myocarditis (epicardial LGE); 4CH cine for LVNC (LV noncompaction in the apex) and 4CH cine for RCM (bi-atrial enlargement on the 4CH view). **b**, ROCs from the diagnostic models based on cine (purple), LGE (yellow) and cine + LGE as combined inputs (blue). Combining cine and LGE yielded the optimal diagnostic performances for all CVD classes. The performance was based on the internal test set.

We further examined the five input schemes: (1) SAX cine, (2) 4CH cine, (3) SAX and 4CH cine, (4) SAX LGE and (5) the combination of SAX cine, 4CH cine and SAX LGE. The all-input scenario achieved the highest AUC and *F*_1_ across all 11 disease classes (Fig. 3 and Extended Data Table 3). We plotted receiver operating characteristic curves (ROCs) for the 11 disease classes. Figure 3 shows the ROCs of three input schemes (cine, LGE and cine + LGE). Notably, the combination of cine and LGE MRIs substantially outperforms models derived from any single modality, with 1.9% points improvement in the averaged AUC metric and 6.8% points improvement in the averaged *F*_1_ metric (compared with SAX cine). All sensitivity and specificity pairs were >90% (Extended Data Table 4). The positive predictive value (PPV) and negative predictive value (NPV) scores are provided in Supplementary Table 1.

### Generalization to external test set

To assess whether our models could be transferred to different institutions with varying data collection protocols, we validated the screening and diagnostic models on external test sets collected from seven medical centers (*n* = 1,819; 403 normal subjects and 1,416 patients with CVDs). Our screening model for anomaly detection attained an AUC of 0.990 (95% CI 0.986–0.992), *F*_1_ score of 0.970 (95% CI 0.964–0.977), sensitivity of 0.959 (95% CI 0.936–0.974) with specificity at 90%, and specificity of 0.970 (95% CI 0.950–0.990) with sensitivity at 90% (Fig. 2 and Extended Data Table 2). The diagnostic model (with all-input scenario) for CVD classification achieved a class-weighted AUC of 0.991 and *F*_1_ score of 0.884 (Fig. 2 and Extended Data Table 5). This indicates that the AI model can generalize across diverse data sources, including medical centers uninvolved during model development.

In addition, we examined the generalizability of models derived from a single imaging modality. The diagnostic models based on cine (SAX and 4CH views) film and LGE achieved cross-institution *F*_1_ scores of 0.831 and 0.792, respectively (Extended Data Table 5). For the screening task, the cross-institution performance was 0.953 (95% CI 0.942–0.965) of AUC by the model derived from SAX cine and 0.980 (95% CI 0.972–0.986) by the model of 4CH cine (Extended Data Table 2). The findings were consistent with that of the primary dataset: the combination of SAX and 4CH cine provides the best performance for detecting cardiac anomalies; integrating cine and LGE yields the optimal diagnostic performance.

### Model interpretability

**Fig. 4:**
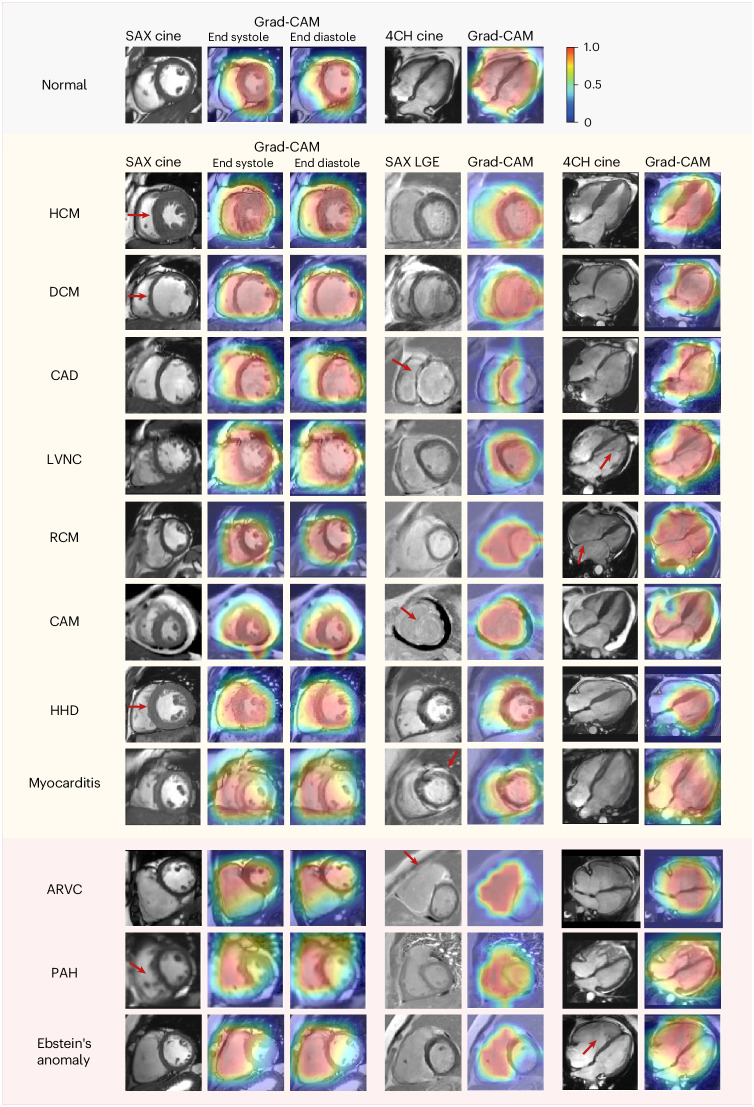
Saliency maps of CMR scans from representative patients of eleven CVD classes and the normal control. The saliency map (heat map) was generated using the guided Grad-CAM approach and reveals the region that contributes the most to the AI model’s decision. The scale bar ranges from zero to one, with one indicating the highest influence provided by the normalized Grad-CAM value and zero indicating the lowest influence. The red arrows point to the characteristic features of each CVD class, which are consistently encompassed by the saliency maps of the diagnostic model: left ventricular hypertrophy, HCM; enlargement of the left ventricle and thinning of the left ventricular wall, DCM; endocardial LGE in the ventricular septum and adjacent anterior of the left ventricular wall, CAD; left ventricular noncompaction in the apex, LVNC; bi-atrial enlargement, RCM; diffuse dust-like LGE of the left ventricular myocardium, CAM; symmetric left ventricular hypertrophy, HHD; subepicardial LGE of the left ventricular free wall, myocarditis; right ventricular enlargement with fibrosis, ARVC; enlargement of the RV and thickening of the right ventricular wall, PAH; apical displacement of the septal valve leaflet of the tricuspid valve, Ebstein’s anomaly. The CVD classes with yellow background are primarily LV dysfunctions and the classes with red background are primarily RV dysfunctions.

We leveraged the guided gradient-weighted class activation mapping (Grad-CAM)^29^ to display an informative set of features and distinct patterns used by the model for classification. Specifically, we extracted the Grad-CAM for representative subjects from 11 CVD categories. Figure 4 shows the AI model activations that contributed to a prediction of CVD. The LV area shows higher saliency at the detection of HCM, DCM, CAD, LVNC, RCM, CAM, HHD and myocarditis (Fig. 4, yellow background); the right ventricle (RV) was highlighted as salient for the detection of ARVC, PAH and Ebstein’s anomaly (Fig. 4, red background). This is consistent with the clinical diagnostic criteria: ARVC, PAH and Ebstein’s anomaly are all primarily RV involvement whereas the abnormality for the rest of the classes is mainly present on LV^30^. In addition, the LGE signal in CAD, CAM, myocarditis and ARVC (Fig. 4, myocardium in SAX LGE, red arrows), which represents myocardial fibrosis or amyloid, was correctly captured by the saliency maps. Furthermore, the model accurately identified the LVNC in the apex and septal leaflet displacement as distinctive features in detecting LVNC and Ebstein’s anomaly (Fig. 4, 4CH cine, red arrows), respectively, which is consistent with the underlying pathophysiology of these conditions^31,32^.

### Comparison with human annotations

To compare the performance of the AI model with that of board-certified physicians, we formed a gold-standard test dataset with 500 patients covering 11 types of CVDs (Extended Data Table 6). Each patient was independently evaluated for CVD class by physicians with three levels of experience in CMR reading (3–5 years, 5–10 years and more than 10 years), along with the AI diagnostic model for comparison (Table 2). The AI model achieved comparable performance with physicians with more than 10 years of experience in CMR reading (*F*_1_ score of 0.931 versus 0.927) with faster speed of interpretation (1.94 min versus 418 min for interpreting 500 subjects). In addition, our model exceeded the performance of the most experienced group of physicians (more than 10 years) for the PAH class by successfully identifying CMR-negative patients (*F*_1_ score of 0.983 versus 0.931). This demonstrates the potential of AI to identify MRI features not readily detectable by humans^33^, a finding consistent with previous works in oncology^34–36^.

**Table 2:** Diagnostic performance of the AI model compared with physicians with varying experience (range from 3 to >10 years) in CMR reading

		No. of subjects (*n* = 500)	*F*_1_ score			
			AI model	Physician (3–5 years)	Physician (5–10 years)	Physician (>10 years)
1	HCM	100	**0.971**	0.957	0.938	0.962
2	DCM	100	0.914	0.853	0.911	**0.940**
3	CAD	80	0.962	0.916	0.949	**0.969**
4	LVNC	30	0.877	0.667	0.778	**0.885**
5	RCM	30	**0.933**	0.578	0.760	0.800
6	CAM	30	**0.947**	0.667	0.931	0.931
7	HHD	30	0.833	0.615	0.667	**0.896**
8	Myocarditis	20	**0.857**	0.553	0.600	0.683
9	ARVC	30	0.897	0.451	0.814	**0.983**
10	PAH	30	**0.983**	0.061	0.929	0.931
11	Ebstein’s anomaly	20	0.950	0.519	0.842	**0.974**
Frequency-weighted *F*_1_			**0.931**	0.734	0.872	0.927
Accuracy			**0.932**	0.746	0.868	0.928
Time cost (in total)			**1.94** **min**	576 min	329 min	418 min

The physicians are categorized according to their number of years of experience in CMR interpretation.

The bold font emphasizes the superior performance metric among subgroups, including the AI model and physicians with varying levels of experience.

### Comparison of video-based deep learning models

**Extended Data Fig. 3:**
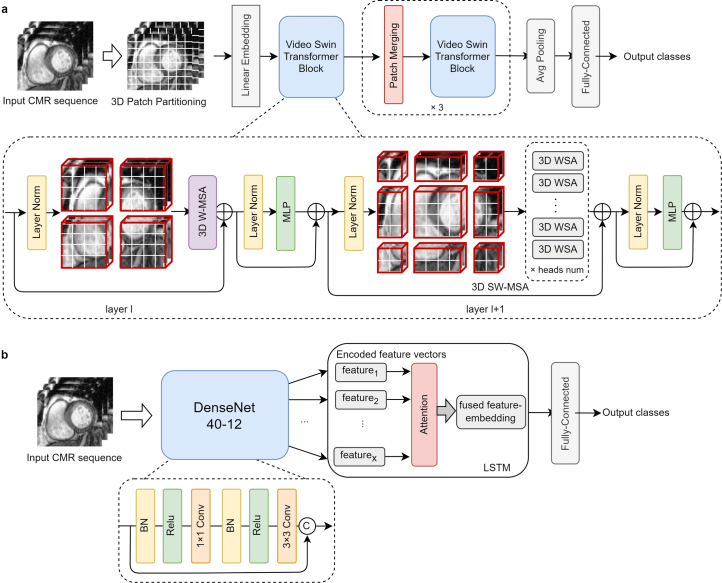
Schematic overview of Video-based Swin Transformer and CNN-LSTM frameworks for CMR interpretation. **a**, the framework of video-based swin transformer (VST). The model takes cardiac MRI sequence as input, extracts distinct features by global self-attention and shifted window mechanism inherent in VST, and outputs the classification score. 3D W-MSA, 3D window based multi-head self-attention module; 3D SW-MSA, 3D-shifted window based multi-head self-attention module; WSA, window self-attention module. **b**, the framework of the conventional CNN-LSTM (Long short-term memory). Each CMR slice is encoded by the DenseNet-40–12 (layer = 40; growth rate = 12) CNN into a feature vector feature i. These feature vectors are sequentially fed into the LSTM encoder, which uses a soft attention layer to learn a weighted average embedding of all slices.

We compared the VST model and the conventional CNN–long short-term memory (LSTM)^21^ approach for modeling CMR sequences. Extended Data Fig. 3 illustrates the schematic overview of the two video-based deep learning algorithms in SAX cine film interpretation. The SAX cine-derived VST model notably outperformed CNN–LSTM with 3.5% points improvement in the AUC and 4.6% points improvement in the *F*_1_ score, tested on the primary dataset. This finding demonstrates the superiority of the VST algorithm in CMR analysis.

### Validation on an independent consecutive test set

To further evaluate the performance of our developed AI model in a real-world clinical setting, we constructed a fresh independent testing set, consisting of 1,000 subjects consecutively admitted to Beijing Fuwai Hospital in 2023. This consecutive testing set was meticulously designed to be unselected, ensuring a representation of the authentic clinical prevalence and encompassing a diverse spectrum of cardiac disease phenotypes.

#### Evaluation of the AI screening model

From the 1,000 consecutively collected subjects, we formed a testing set for the screening model comprising 961 subjects with complete cine images, including 159 normal individuals and 802 patients with cardiac anomalies. Thirty-nine subjects were excluded based on the following criteria: (1) missing SAX cine or 4CH cine sequences (22 subjects), (2) SAX cine with fewer than five views (six subjects) and (3) inadequate imaging quality (11 subjects). Utilizing cine MRI from both SAX and 4CH views, the AI screening model demonstrated exceptional performance on the independent consecutive testing set (*n* = 961; Supplementary Table 2), achieving an AUC of 0.984 (95% CI 0.977–0.990) and an *F*_1_ score of 0.962 (95% CI 0.953–0.972) for cardiac anomaly screening. The sensitivity of 0.946 (95% CI 0.930–0.964) was achieved by the screening model for cardiac anomaly detection with specificity at 90%. The screening model performance is detailed in Supplementary Table 2. Notably, the consecutive testing set encompassed a diverse range of CVDs, including mild/borderline cases and suspected phenocopies (for example, inherited metabolic cardiomyopathies), extending beyond the commonly identified 11 CVD classes. This underscores the robustness of the screening model with respect to both disease types and severity.

#### Evaluation of the AI diagnostic model

From the 1,000 consecutively collected subjects, we formed a testing set for the diagnostic model, comprising 532 patients with CVD and complete sets of LGE and cine images. To ensure the integrity of the testing set, we established detailed exclusion criteria. Specifically, 159 normal individuals without cardiac anomalies were excluded, along with 222 patients lacking LGE images, which are essential inputs for our diagnostic model. It is crucial to note that LGE, an invasive examination requiring contrast injection, was not consistently performed for all admitted patients. Additionally, 48 patients with CVD, falling beyond the scope of the commonly identified 11 CVD classes, were excluded from the reported quantitative testing performance. Nevertheless, we have included and analyzed the AI screening and diagnostic results for these 48 patients in Supplementary Table 3.

With the established testing set (*n* = 532), our AI diagnostic model, utilizing cine and LGE images as combined inputs, demonstrated exceptional performance. It achieved a class-weighted average AUC of 0.986 and an *F*_1_ score of 0.903 (Supplementary Table 4). Notably, the model exhibited high AUCs and *F*_1_ scores for prevalent CVDs, including HCM (AUC 0.993, 95% CI 0.988–0.997; *F*_1_ 0.958, 95% CI 0.940–0.975), DCM (AUC 0.991, 95% CI 0.983–0.996; *F*_1_ 0.922, 95% CI 0.883–0.958) and CAD (AUC 0.997, 95% CI 0.994–0.999; *F*_1_ 0.915, 95% CI 0.855–0.966). Across all 11 CVD classes, the model achieved an AUC greater than 0.90, with *F*_1_ scores above 0.80 for all except LVNC, HHD, RCM and myocarditis. The CAM class exhibited a high *F*_1_ score of 0.947 and an AUC of 1.0.

## Discussion

CMR has been considered the gold standard for assessing cardiac function; its contemporary application encompasses virtually all aspects of CVDs. It shows unique capabilities in the diagnostic workup of suspected CVD^37^. However, CMR is also one of the most challenging radiologic imaging techniques to interpret due to the complexity of cardiac motion. In this study, we conducted a pioneering investigation in computerized CMR (cine and LGE) interpretation for screening and diagnostics. Our study of 8,066 patients with CVD and 1,653 normal individuals concluded that the screening model for anomaly detection and diagnostic model for CVD classification attained AUCs of 0.988 ± 0.3% and 0.991 ± 0.0% (*F*_1_ scores of 0.974 ± 0.5% and 0.895 ± 1.6%; mean ± s.d. of internal set and external set), respectively. These results demonstrate that video-based end-to-end deep learning approaches can reliably detect anomalies and classify various types of CVDs from CMR with high classification performance similar to or even superior to that of experienced cardiologists.

This proof-of-concept study shows an automatic pathway to CMR analysis. The standard clinical approach to CMR interpretation requires experts to (1) manually delineate the contours of the endocardium and epicardium and (2) scan back and forth across cine film and LGE over a series of SAX and long-axis views. Specifically, a typical CMR examination consists of SAX cine films with nine parallel views (25 frames per view), a 4CH cine film (25 frames), a three-chamber cine film (25 frames), SAX LGEs (nine parallel views) and 4CH LGE, leading to at least 11 videos and 10 images to analyze in total. Hence, this procedure is extremely labor intensive, time consuming and susceptible to operator bias. In contrast, deep neural networks (DNNs) enable an approach that is fundamentally different since the automatic model can absorb all pieces of information present in CMR ‘end-to-end’ without requiring manual tracing, calculation of cardiac function or class-specific feature extraction. In other words, the proposed DNN model accepts the raw CMR data as input, learns all of the important features—both previously manually derived and as-yet-unrecognized—in a data-driven way and outputs final diagnostic probabilities.

The high performance of the developed screening models derived from cine MRI suggests a fast, noninvasive and accurate screening technique for detecting CVDs. The screening model derived from 4CH cine achieved an AUC of 0.977 ± 0.4% (mean ± s.d. of internal set and external set; Extended Data Table 2); the model derived from SAX cine achieved an AUC of 0.962 ± 1.3%. The single-view schemes yielded similar performance as combined views (the model derived from 4CH and SAX cine received an AUC of 0.988 ± 0.3%). Therefore, the finding that a single view can independently and reliably detect cardiac anomalies indicates that this method can be used to simplify CMR acquisition and improve clinical efficiency. Increased efficiency is beneficial, given the potential to decrease the cost of cine MRI acquisition and enhance patient throughput. The shortened procedure time is also beneficial for patients who cannot tolerate longer scans. In addition, cine MRI provides high-resolution images for accurate quantitation of ventricular volume, cardiac function and motion estimation, along with detailed signals in myocardium, which together form the cornerstone of diagnosis^38^. As such, the cine-based screening test can serve to improve the accuracy of anomaly detection in CVD, particularly since there is ample evidence to suggest that the most widely used screening examinations—electrocardiogram (ECG) and echocardiogram—capture only a fraction of the informative features for diagnosis^39,40^.

CVD diagnosis is one of the most problematic and challenging tasks in cardiology. To address the challenge, this study introduced automatic diagnosis based on CMR. Cine and LGE MRIs together substantially outperformed the model derived from either cine or LGE alone. This finding is consistent with prior studies demonstrating that cine and LGE provide complementary information in CMR diagnosis^41^. The diagnostic model derived from cine and LGE yielded an average class-weighted AUC of 0.991 over 11 classes. The 11 classes account for most of the CVDs referred for CMR examination (over 90% at Beijing Fuwai Hospital^28^), making the model broadly applicable. This outcome effectively propels us toward making efficient and precise CVD diagnosis that has a significant clinical impact. As provider confirmation will still be needed in many clinical settings and ambiguous cases, we expect the diagnosis model to complement, not replace, cardiologists. The AI model could expand the capability of a CMR-trained cardiologist in the clinical workflow by triaging the readings for which the model has the least ‘confidence’.

Moreover, the AI model’s ability to outperform cardiologists in diagnosing PAH by successfully identifying CMR-negative cases (that is, confirmed PAH without abnormal CMR findings) can have marked clinical impact by allowing for less invasive diagnosis of PAH. PAH is a progressive condition with high mortality, and timely diagnosis is vital for its treatment^42^. The current gold standard for diagnosis of PAH is right heart catheterization, which is an invasive procedure that can introduce serious surgical complications including hematoma, pneumothorax, arrhythmias and hypotensive episodes^43–45^. The conventional CMR evaluation of the RV has been used to assess the severity of PAH and monitor its prognosis and therapy response^46^. While CMR’s diagnostic utility in PAH is largely underexplored due to its technical complexity^47^, the AI-empowered CMR interpretation demonstrated in this study offers a timely and valuable perspective and pathway for an accurate, safe and rapid PAH diagnosis.

Of the CVD classes we examined, myocarditis is a clinically important CVD for which the diagnostic model derived from cine and LGE had a lower *F*_1_ score compared with other CVD classes (internal set: 0.724; external set: 0.630). A manual review of the discordances revealed that the model misclassifications overall appear very reasonable. For example, some instances of mild myocarditis only present mild elevation of troponin with no remarkable myocardial necrosis, leading to an LGE-negative result. Meanwhile, the edema and functional ventricular impairment could be relieved if patients with myocarditis are not scanned in the appropriate time window, resulting in CMR negativity. This is consistent with the general findings: the sensitivity of myocarditis diagnosis based on the Lake Louise criteria—the diagnostic CMR imaging criteria for patients with suspected myocarditis—only reaches 0.780–0.875 (refs. ^48,49^). Moreover, for myocarditis diagnosis, the lack of T2-weighted images and parametric myocardial mapping^50^ limited the conclusions that could reasonably be drawn from the cine and LGE MRI, making it more difficult to definitively ascertain whether the cardiologists and/or the AI model was correct.

We emphasize our use in this study of a CMR dataset representative enough (covering a wide spectrum of 11 types of CVDs, accounting for above 90% of the CVD patients referred for CMR examination and CMR acquired by three major vendors) to evaluate end-to-end deep learning approaches for screening and diagnostics and our comprehensive internal and external validations of 9,719 subjects pooled from eight medical centers. We leveraged more than one million cardiac MRI images comprising 38,876 cine films and 72,594 LGE images. To the best of our knowledge, large pooled CMR databases containing both cine and LGE modalities that can be used to diagnose a wide range of heart conditions do not currently exist. As such, our collected cohort is unique in that it is the largest and first-ever complete CMR database with cine and LGE MRIs for AI-enabled studies.

We leveraged VST as our model backbone of choice in CMR interpretation. Transformer-based deep learning architectures very recently expanded to image and video processing and yield substantial improvements on a wide spectrum of high-level computer vision tasks. VST, a transformer adapted for video sequence processing, has shown impressive performance on the major video recognition benchmarks^27^. However, few efforts have been made to explore its role in medical video analysis. As opposed to the conventional CNNs, which are limited by the small receptive field of the convolution operation, the global self-attention and shifted window mechanism inherent in VST broadens the receptive field and allows effective integration of temporal and spatial information from cardiac video and three-dimensional (3D) sequences. The superiority of VST confirmed in this study offers insight into the use of AI-enabled medical video analysis within and beyond CMR imaging.

Several limitations need to be considered when interpreting the presented results. Extensive evaluation through prospective studies and clinical trials is necessary before the models’ clinical implementation. The reported algorithmic performance may not translate to real-world deployment, necessitating further validation. All participating institutions are from eastern Asia. The model generalizability across different ethnicities should be investigated in future work to ensure its broad utility. The number of health controls was limited compared with the overall study population. Owing to this, a more comprehensive assessment of the screening model based on a dataset with real-world CVD prevalence is warranted. While the screening model demonstrates robustness in handling abnormal cases outside the specified 11 commonly encountered CVD classes, the diagnostic model’s ability to distinguish cases with unique phenocopies, such as Fabry disease, inherited metabolic cardiomyopathies and instances of dual conditions, stands as a pivotal focus for future research. A potential solution involves the integration of a deferral AI^51^, leveraging the synergies between human clinicians and AI models within the predictive system to further augment the reliability of AI-empowered CMR-based diagnosis. Notably, enhanced diagnostic model performance can be anticipated by integrating patients’ clinical history and CMR imaging, which should be a focus of forthcoming endeavors. For example, the inclusion of pertinent clinical factors such as a prolonged history of arterial hypertension could effectively aid in distinguishing between HHD and DCM^52,53^, especially in cases where their CMRs exhibit similar characteristic features. In the present study, our focus was limited to cine and LGE modalities. Future research should include quantitative T1 and T2 mapping, as well as extracellular volume fraction data, due to their diagnostic relevance in CVD^54,55^. Lastly, additional studies focusing on deep learning model interpretability are needed. The Grad-CAM findings in Fig. 4 further demonstrate the validity of the models’ CMR interpretation. Nonetheless, it is not sufficient and full interpretability will be a focus of future work.

In summary, we demonstrate that end-to-end video-based deep learning models can detect cardiac anomalies and further classify distinct CVDs from CMR with high classification performance. If confirmed in clinical settings, our study has the potential to substantially advance the efficiency and scalability of CMR interpretation, paving the way for widespread use of CMR in CVD screening and diagnosis.

## Methods

### Ethics approval

The CMR datasets were acquired retrospectively under the approval of the institutional review boards (IRBs) at each participating institution, including Beijing Fuwai Hospital, Beijing Anzhen Hospital, Guangdong Provincial People’s Hospital, the 2nd Affiliated Hospital of Harbin Medical University, the First Hospital of Lanzhou University, Renji Hospital, Tongji Hospital and Peking Union Medical College Hospital. Informed consent was waived by the IRBs. Before model training, testing and reader studies, all data underwent deidentification processes.

### Datasets

The CMR database search was performed for all eight centers to identify CVDs and normal controls. All data were anonymized and deidentified, as per the Health Insurance Portability and Accountability Act Safe Harbor provision^56^. Inclusion criteria were (1) patients with a definitive diagnosis of CVD and (2) patients with CMR scans at baseline before surgical treatment, if any. Exclusion criteria were (1) incomplete cine or LGE modalities, (2) SAX cine with fewer than five views, (3) CMR images with insufficient scan quality, (4) CVD patients missing clinical data and (5) CMR examinations that could not be interpreted and agreed upon by the committee cardiologists according to the diagnostic criteria (Methods). The detailed diagnostic criteria of the 11 types of CVDs and normal controls included in this study was described in Methods. Table 1 and Extended Data Table 1 present the detailed demographics and distribution of the primary dataset and the external validation sets collected from the other seven medical centers across China. To offer a comprehensive perspective on our primary development dataset, we went the extra mile by collecting the LV ejection fraction (LVEF) metric for all 7,900 subjects (including 1,250 normal controls and 6,650 patients with CVD) within the primary dataset. We meticulously summarized the distribution of demographics and LVEF across the 11 specified CVD classes and the normal control class in Supplementary Table 5. Additionally, we generated density plots to illustrate the distribution of LVEF for each class in the primary dataset, offering a more comprehensive representation (Supplementary Fig. 1).

The fresh consecutive testing set is designed to capture the genuine spectrum of disease phenotypes in the real-world clinical prevalence. To offer a thorough understanding of the severity of cases in alignment with real-world clinical prevalence, we have presented five key cardiac function metrics. These metrics include LVEF, LV mass, LVMi (LV mass index), LV end-diastolic volume and LV end-diastolic volume index. Supplementary Table 6 presents the distribution of demographics and the cardiac functions across 11 CVD classes and the normal control class in the fresh consecutive testing set. For improved visualization and clarity, we have depicted the prevalence of the 11 CVD classes in both the fresh consecutive testing set (*n* = 532 patients with CVD) and the primary discovery dataset (*n* = 6,650 patients with CVD) using pie charts in Supplementary Fig. 2. The fresh consecutive testing set offers a representation of the genuine clinical prevalence. Through direct comparison, it is evident that the primary dataset and the consecutive testing set exhibit very similar CVD prevalence and distribution. The top three most prevalent CVDs referred to the CMR examination remain HCM, DCM and CAD.

All images were acquired by breath-holding and electrocardiographic gating. A balanced steady-state free precession sequence was used for cine images with a continuous sampling from the basal to the apical levels on SAX views and two-chamber, three-chamber and 4CH long-axis views. We included cine MRI from two views in this study: the standard SAX cine and the long-axis 4CH cine. The SAX cine clearly depicts the RV and the LV. The 4CH cine shows the four chambers of heart: right atrium, left atrium, RV and LV.

LGE MRI has been established as the gold standard reference for myocardial viability and replacement fibrosis in the myocardium^57,58^. In our CMR cohorts, the LGE images were obtained using phase-sensitive inversion recovery sequence with a segmented FLASH readout scheme performed 10–15 min after injection of gadolinium-based contrast with 0.15 mmol kg^−1^ per bolus. Gadolinium contrast agents can be used to detect areas of fibrosis, as the prolonged washout of the contrast correlates with a reduction in functional capillary density in the irreversibly injured myocardium^59^. The SAX LGE used in the study was acquired from the SAX view with the same section thickness, covering the entire left ventricle from the base to the apex (nine parallel views for most cases). Note that LGE is an invasive examination that requires contrast injection and was therefore not performed for normal controls.

The typical CMR scan protocol and scanner parameters for the primary and external validation sets are presented in Supplementary Table 7. Extended Data Fig. 2 shows an illustration of cardiac MRIs (SAX cine, 4CH cine and SAX LGE) utilized in model development. Supplementary Videos 1–11 demonstrate example CMR of the 11 types of CVDs.

### Annotation procedures

For each patient in the disease cohort, the textual description of the abnormalities in the CMR and the clinical report was extracted as the main reference. Besides that, all CMR records underwent additional annotation procedures. To annotate the disease cohort, a group of certified CMR experts reviewed all records and clinical reports. Every record was randomly assigned to be reviewed by a single physician specifically for this task, not for any other purpose. All annotators received specific instructions and training regarding how to annotate CMR data to improve labeling consistency. The diagnostic criteria we adopted in this study for each CVD class are described in Methods. CMR examinations that could not be interpreted by physicians received further annotation from a consensus committee of board-certified practicing cardiologists (with >15 years of experience in CMR reading) working in Fuwai Hospital. The CMR examinations that could not be interpreted or agreed upon by the committee were removed from our dataset.

For the independent gold-standard test dataset with 500 patients (Extended Data Table 6) for human–machine comparison, six physicians working in the MRI department at Fuwai Hospital contributed directly to its annotation (the six physicians were not involved in dataset annotation as described above). All participating physicians received specific instructions and training regarding how to annotate CMRs to ensure consistency. We divided the physicians into three groups according to their reading experience in CMR: 3–5 years, 5–10 years and more than 10 years. CMR physicians in each group reviewed a randomly selected set of the 500 CMRs in a nonrepetitive manner.

### CMR preprocessing

**Extended Data Fig. 4:**
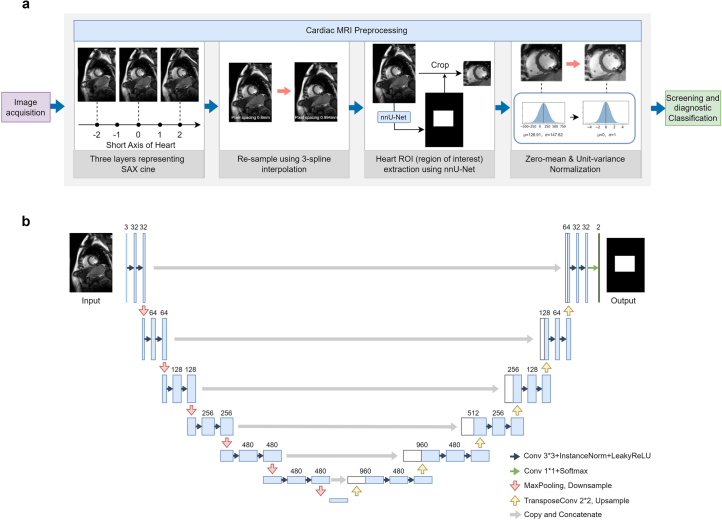
The preprocessing pipeline and the framework of heart region of interest (ROI) automated detection. **a**, the preprocessing pipeline for SAX cine. **b**, the framework of ROI detection for SAX cine. The model takes a single cine sequence as the input and outputs the bounding box (binary mask) covering the heart ROI. SAX, short-axis; ROI, region of interest.

The CMR preprocessing pipeline aimed to remove the additional burden of the deep neural network learning to find patterns between images for disease classification. All cardiac MRIs were preprocessed to (1) resample MRI images to the same spatial resolution and (2) localize the heart region of interest (ROI) to a crop image. We detailed the preprocessing step for cine and LGE MRI below and in Extended Data Fig. 4.

SAX cine comprises nine parallel views (for most cases) covering the apical to the basal levels of the LV. Each view contains 25 frames (cardiac phases), leading to 225 images in one single SAX cine record. We examined the representational power of different numbers of input views in developing the classification model. Balancing efficiency and effectiveness, the three-view input scheme achieved a greater representation of SAX cine and therefore is adopted throughout the rest of the study. The three-view input scheme includes the middle layer (the mid slice among the parallel layers spanning from the base to the apex), the second layer above the middle layer and the second layer below the middle layer (Extended Data Fig. 2). We extract the ‘ImagePositionPatient’ tag and the ‘ImageOrientationPatient’ tag from each Dicom header to locate the three layers. Then, three-spline interpolation provided by SimpleITK^60^ library (https://simpleitk.org/) is applied to resample the raw cine MRIs to the same spatial resolution of 0.994 mm × 0.994 mm, which is the most common spatial resolution across all subjects investigated in this study. We developed a heart ROI segmentation model (the following section) and used it to localize the region of heart for each cine MRI. The heart ROI segmentations predicted by the AI models were manually checked to ensure their accuracy. The extracted ROIs are padded to keep the aspect ratio the same without distortion, and then resized to 224 × 224. The top and bottom 0.1% of the pixels in cine MRI images are clipped to avoid pixels that are outliners of the distribution. The cine images are scaled between 1 and 255, and then normalized by zero mean and unit variance before feeding them to the model. We sample a clip of 25 frames from each full-length cine sequence using a temporal stride of two, resulting in 13 frames as inputs to model development. The 4CH cine shares the same preprocessing pipeline as SAX cine, except that only one single layer (mid slice) is used to represent the 4CH view. For SAX LGE, all layers covering from the base to the apex of the heart are used for diagnostic model development. The preprocessing steps for SAX LGE are similar to that of cine MRI. We resampled SAX LGE along the *z*-axis to ensure that each LGE sequence contains nine slices because nine is the most common number of views for SAX LGE included in this study.

### Heart ROI extraction

We developed heart detection DNN models to automatically extract the heart ROI regions (Extended Data Fig. 4). Three DNN models for SAX cine, 4CH cine and SAX LGE were trained and evaluated, respectively. We applied nnU-Net^61^ as our model backbone and generated the ground-truth segmentation masks for model supervision using a semi-automatic approach. (1) Automatic localization: for SAX cine and 4CH cine, we selected the pixel region with maximum standard deviation across all frames. These regions localize the heart ROI as heart is a beating organ with high standard deviation in its position. Specifically, for each cine movie sequence $s=\{{x}_{1},\ldots ,{x}_{n}\}$, we computed a single pixel map of standard deviations across all frames ${x}_{\mathrm{std}}=\sigma (\{{x}_{1},\ldots ,{x}_{n}\})$. This map was used to compute an Otsu threshold to binarize and label regions with the greatest variation in cine modality^21^. For each cine sequence, a binary segmentation mask of the heart ROI is defined for the length of the cardiac cycle. All segmentation masks went through manual checking. The localization procedure captures the heart ROI in around 90% of cases. The rest of the cases are labeled manually. (2) Manual labeling: we manually drew the bounding box capturing the heart ROI, using 3D Slicer^62^ and ITK-SNAP^63^. We used the Scissors tool provided by the Segment Editor in 3D Slicer and the Polygon Inspector in ITK-SNAP to locate heart ROI. A binary segmentation mask was saved for each CMR sequence. For SAX LGE, we manually drew the annotations as model supervision.

In terms of model architecture, the detection model shares the classic U-net^64^ backbone with three small adjustments: (1) batch normalization is replaced with instance normalization^65^, (2) rectified linear unit (ReLU) is replaced with leaky ReLU^66^ as the activation function and (3) additional auxiliary losses are added in the decoder to all but the two lowest resolutions. The model outputs the binary bounding box that extracts the heart ROI. For model training, we adopted Adam optimizer and stochastic gradient descent (SGD) with Nesterov momentum (*μ* = 0.99). The initial learning rate was set to be 0.01, and the decay of the learning rate followed the ‘Poly’ learning rate policy^67^. Batch size was set to 36. Data augmentation included rotations, scaling, gamma correction and mirroring. The loss function is the sum of cross-entropy and Dice loss^68^.

### Video-based deep learning models and training details

#### Model architecture

For models based on cine sequence, we sampled a clip of 13 frames from each 25-frame cine video using a temporal stride of 2 and spatial size of 224 × 224, resulting in 7 × 56 × 56 input 3D tokens. The 3D patch partitioning layer obtains tokens, with each patch/token consisting of a 128-dimensional feature. In practice, 3D convolution without overlapping is applied for this tokenization, and the number of output channels is set to be 128 to project the features of each token to a 128-dimension.

The developed model consists of four stages, that is, four video swin transformer blocks. Each stage, besides the last stage, performs 2× spatial downsampling in the patch merging layer. It is worth noting that we do not downsample along the temporal dimension. The patch merging layer concatenates the features of each group of 2 × 2 spatially neighboring patches and applies a linear layer to project the concatenated features to half of their dimension. The video swin transformer block consists of a 3D window-based multihead self-attention module and a 3D-shifted window-based multihead self-attention module, followed by a feedforward network, that is, a two-layer multilayer perceptron, with Gaussian error linear unit nonlinearity in between. Layer normalization is applied before each multihead self-attention module and multilayer perceptron, and a residual connection is applied after each module. We used the base version of VST. The number of heads for each stage is 4, 8, 16 and 32. Extended Data Fig. 3a shows the schematic overview of the VST-based framework for modeling SAX cine.

#### Data augmentation

Model performance improved with increasing training data sample size. For the screening model, we used random rotation, random color jitter and adding random number. During each step of SGD in the training process, we perturbed each training sample, cine video sequences, with a random rotation (between −45 and +45 degrees for SAX cine and between −20 to +20 degrees for 4CH cine), random color jitter and with adding a number sampled uniformly between −0.1 and 0.1 to image pixels (pixel values are normalized) to increase or decrease brightness of the images. For LGE, we used random rotation between −45 and +45 degrees, random color jitter and random flip along the *z*-axis. Data augmentation resulted in improvement for all models.

#### Multimodality fusion

First, we developed VST-based models for SAX cine, 4CH cine and SAX LGE, respectively. Then, to fuse information from different modalities, we added a global average pooling layer following the last self-attention module for each VST model. This resulted in a 1,024-dimension feature vector from each modality. We further concatenated the 1,024-dimension vectors and added a fully connected layer on top of that to aggregate the features. The final fully connected softmax layer produces a distribution over the output classes. In terms of training, we loaded and froze the pretrained weights of each VST branch from different modalities using transfer learning^69^ and only finetuned the last fully connected layers for feature aggregation.

#### Implementation details

Following the classic VST configuration^27^, we employed an AdamW optimizer using a cosine decay learning rate scheduler and 2.5 epochs of linear warmup. A batch size of 32 was used. The backbone VST is initialized from the ImageNet^70^ and Kinetics-600 (ref. ^71^) pretrained model; the head is randomly initialized. Model pretraining plays a strikingly important role in VST-based CMR interpretation. We also found that multiplying the learning rate of the backbone by 0.1 improves performance. Specifically, the initial learning rates for the pretrained backbone and randomly initialized head were set to be 1 × 10^−4^ and 1 × 10^−3^, respectively. The impact of learning rate modification on the VST backbone was systematically examined as below. We adopt 0.2 stochastic depth rate and 0.05 weight decay for the Swin base model used in this study. To prevent the models from becoming biased toward one class, we balanced the training datasets for both screening and diagnostics using the ClassBalancedDataset sampling strategy^72^. Each VST branch derived from the single modality was trained for 150 epochs and then fed into the fusion model, following with 20 epochs of finetuning particularly for the fusion layers. For inference, we set the batch size to be one and the number of workers to be four. The training time for model development using four NVIDIA GeForce RTX 3090 graphics processing units with 24 GB VRAM was about 77 h, and the inference time for each subject was only 0.233 s.

#### Learning rate on the VST backbone

The impact of learning rate modification on the VST backbone was systematically examined through a controlled experiment. The experiment encompassed a range of learning rates, from 1 × 10^−2^ to 1 × 10^−6^, with a focus on their effects on the AI diagnostic model based on SAX cine. The investigation was conducted on the primary cohort (6,650 CVD patients), utilizing a twofold configuration for training and the remaining fold for testing. The model was trained for 150 epochs with five different learning rate initializations for the model backbone: 1 × 10^−2^, 1 × 10^−3^, 1 × 10^−4^ (as applied in this study), 1 × 10^−5^ and 1 × 10^−6^. Other configurations were kept consistent for a fair and direct comparison, and the training loss for each scheme was plotted for analysis (Supplementary Fig. 3). From the depicted figure, several key observations emerge. When the learning rate is set too high (1 × 10^−2^, curve in blue color), the model struggles to converge and the training loss fails to descend, in stark contrast to the more optimal setting of 1 × 10^−4^ (curve in green color). Notably, the model under the 1 × 10^−2^ learning rate incorrectly classified all samples into the HCM class during testing. Conversely, when the learning rate is set too low (1 × 10^−6^, curve in purple color), the loss descends very slowly over the training period. As depicted in the figure, the loss curves for 1 × 10^−5^ and 1 × 10^−6^ remain at a relatively high level compared with the more effective setting of 1 × 10^−4^. Further evaluation included the calculation of *F*_1_ and area under the receiver operating characteristic curve scores for the testing fold under the aforementioned experimental settings (Supplementary Fig. 3). Notably, the model trained with a learning rate of 1 × 10^−2^ failed to converge and was consequently excluded from the quantitative metrics. According to the evaluation results, the initialized learning rate of 1 × 10^−4^ demonstrated superior performance compared with the other settings. Therefore, based on these comprehensive analyses, we selected 1 × 10^−4^ as the initialized learning rate for our experiment.

#### CNN–LSTM

We examined the conventional CNN–LSTM architecture in CMR interpretation. The CNN–LSTM consists of a DenseNet encoder with 40 layers and a growth rate of 12 for feature extraction and an LSTM for temporal feature aggregation. DenseNet encoder comprised a series of two-dimensional convolutions with kernel sizes 1 × 1 and 3 × 3 and global average pooling to extract the feature vector for each input frame. For LSTM, the feature vector for each input frame is fed into the LSTM module sequentially. LSTM fuses the feature vectors and produces the final classification score after one fully connected layer. For the training configuration of the CNN–LSTM model, we adopt the SGD optimizer with a learning rate of 0.001, a momentum of 0.9 and a weight decay of 0.001. A batch size of four is used for training and one is used for testing. The DenseNet encoder of the CNN–LSTM model is initialized from the pretrained model^21^ and the LSTM component is randomly initialized. We kept data augmentation, the input scheme and computational resources the same as VST models with the only difference: SAX cine inputs are resized to 64 × 64 due to CNN–LSTM memory constraints.

### Quantitative assessment and statistical analysis

The performance of the AI models was evaluated by assessing their sensitivity, specificity, precision and *F*_1_ score (harmonic mean of the predictive positive value and sensitivity), with two-sided 95% CIs, as well as the AUC of the ROC with two-sided CIs. The *F*_1_ score is complementary to the AUC, which is particularly useful in the setting of multiclass prediction and less sensitive than the AUC in settings of class imbalance. For an aggregate measure of model performance, we computed the class frequency-weighted mean for the *F*_1_ score and the AUC^73^.

The cutoff value was set to 0.5 for screening; the CVD class with the highest probability was the diagnostic prediction. Precision, sensitivity (recall), specificity, PPV, NPV and *F*_1_ score of each class are related to true-positive (TP), true-negative (TN), false-positive (FP) and false-negative (FN) rates, with formulas as follows:

$$\text{Sensitivity}=\frac{\mathrm{TP}}{\mathrm{TP}+\mathrm{FN}},$$

$$\text{Specificity}=\frac{\mathrm{TN}}{\mathrm{TN}+\mathrm{FP}},$$

$$\mathrm{Precision}=\frac{\mathrm{TP}}{\mathrm{TP}+\mathrm{FP}},$$

$$\mathrm{PPV}\,=\frac{\mathrm{TP}\,}{\mathrm{TP}+\mathrm{FP}},$$

$$\mathrm{NPV}\,=\frac{\mathrm{TN}\,}{\mathrm{TN}+\mathrm{FN}},$$

$${F}_{1}\text{-score}=\frac{2\times \mathrm{Precision}\times \mathrm{Sensitivity}}{\mathrm{Precision}+\mathrm{Sensitivity}}.$$

The ROC space is defined by 1 – specificity and sensitivity as the *x* axis and the *y* axis, respectively. It depicts relative trade-offs between true positive and false positive, as the classification threshold goes from zero to one. A random guess will give a point along the diagonal line from the bottom left to the top right. Points above the diagonal line represent good classification results and points below the line represent bad results. We applied the class frequency-weighted *F*_1_ score and class frequency-weighted AUC to evaluate the performance of our diagnostic model, with the following formulas:

$${\rm{Weighted}}\,{F}_{1}\text{-}{\rm{score}}=\mathop{\sum }\limits_{i}^{C}{\mathrm{ratio}}_{i}{F}_{1}\text{-}{\mathrm{score}}_{i},$$

$${\rm{Weighted}}\,\mathrm{AUC}=\mathop{\sum }\limits_{i}^{C}{\mathrm{ratio}}_{i}{\mathrm{AUC}}_{i},$$

where ${{F}_{1}\text{-score}}_{i}$ and AUC_*i*_ denote the *F*_1_ score and AUC for class *i*, respectively, and ${\mathrm{ratio}}_{i}$ denotes a frequency ratio for each class *i*.

In addition, to improve the model interpretability and visualize the features used by the DNN model that determine the final prediction, we used Grad-CAM^29^ to localize important regions—saliency regions—by visualizing class-specific gradient information. In Grad-CAM, the neuron importance weight ${\alpha }_{k}^{\,c}$ is estimated as

$${\alpha }_{k}^{\,c}=\frac{1}{Z}\sum _{i}\sum _{j}\frac{\partial {y}^{\,c}}{\partial {A}_{{ij}}^{k}},$$

where *y*^*c*^ denotes the gradient score for class $c$ before the softmax and *A*^*k*^ denotes the feature map activation of the *k*th layer. After computing the neuron importance weights for each feature map, we can generate a heat map indicating the significant regions related to class $c$ by performing a weighted linear combination of the feature maps, followed with a ReLU activation function as

$${L}_{\mathrm{Grad}-\mathrm{CAM}}^{c}=\mathrm{ReLU}\left(\sum _{k}{\alpha }_{k}^{\,c}{A}^{k}\right).$$

We then used the Shapley values^74^ to evaluate the influence of each input modality (SAX cine, 4CH cine and SAX LGE). The Shapley value is a principled attribution method used in AI to quantify the contribution of individual input features by assigning each input modality an importance value for a particular prediction. The definition of the Shapley value^75^ is given in equations below:

$${{{\phi }}}_{i}\left(v\right)=\sum _{S\subset N\{i\}}{\left(\begin{array}{c}n\\ 1,\left|S\right|,n-\left|S\right|-1\end{array}\right)}^{-1}\left(v\left(S\cup \{i\}\right)-v\left(S\right)\right),$$

where ${\phi}_{i}\left(v\right)$ denotes the contribution value of input component *i*, namely the Shapley value of each input modality (player), $N$ is the number of layers and $v$ is a function mapping subsets of layers to the real numbers: $v:{2}^{N}\to {R}$, with $v\left(\varnothing \right)=0$, where $\varnothing$ denotes the empty set. A set of players is called a coalition. The function $v$ is called a characteristic function: if $S$ is a coalition of players, then $v(S)$, called the worth of coalition $S$, describes the total expected sum of payoffs the members of $S$ can obtain by cooperation. The sum extends over all subsets $S$ of $N$ not containing input component *i*; also note that $$\left(\begin{array}{c}n\\ a,{b},{c}\end{array}\right)$$ is the multinomial coefficient. This formula can also be interpreted as

$$\begin{array}{l}{{{\phi }}}_{i}\left(v\right)=\frac{1}{{\mathrm{Number}}\;{\rm{of}}\;{\rm{layers}}}\\\sum _{{\mathrm{coalitions}}\; {\mathrm{including}}\;i}\frac{{\mathrm{Marginal}}\;{\mathrm{contribution}}\; {\mathrm{of}}\;i\;{\mathrm{to}}\;{\mathrm{coalition}}}{{\mathrm{Number}}\; {\mathrm{of}}\; {\mathrm{coalitions}}\; {\mathrm{excluding}}\;i\; {\mathrm{of}}\; {\mathrm{this}}\; {\mathrm{size}}}.\end{array}$$

### Diagnostic criteria of the CVDs and normal control

#### CAD or ischemic cardiomyopathy

The diagnosis of myocardial infarction or ischemic cardiomyopathy is based on the European Society of Cardiology, American College of Cardiology and American Heart Association committee criteria^76^ with significant stenosis on invasive coronary angiography (CAG) or coronary computed tomography angiography, and CMR showed subendocardial or transmural LGE with matching coronary arteries. We excluded cases without available CAG present or inadequate image quality due to arrhythmia or respiratory motion artifact.

#### HCM

We followed the 2020 American Heart Association and American College of Cardiology guidelines for the diagnosis of patients with HCM^77^. The clinical diagnosis of HCM was made by CMR showing a maximal end-diastolic wall thickness of ≥15 mm anywhere in the LV, in the absence of another cause of hypertrophy in adults. More limited hypertrophy (13–14 mm) can be diagnostic when present in family members of a patient with HCM or in conjunction with a positive genetic test.

We excluded cases with the following conditions:

1. Valvular heart disease (aortic valve stenosis, etc.)
2. Long-term uncontrolled hypertension
3. Inflammatory heart disease (sarcoidosis, etc.)
4. Infiltrative cardiomyopathy (amyloidosis, Fabry disease, etc.)
5. Septal myectomy or alcohol ablation before CMR
6. CMR images with poor quality

#### DCM

The diagnosis of DCM is based on the diagnostic criteria of the World Health Organization^78^. Inclusion criteria were based on enlarged LV end-diastolic dimension (>60 mm) and reduced LVEF (<45%). The exclusion criteria were as follows:

1. Significant stenosis of coronary artery (>50% stenosis, assessed on CAG or coronary computed tomography angiography)
2. Severe valvular disease, hypertension or congenital heart disease
3. Evidence of acute or subacute myocarditis (T2 weighted image and laboratory tests)
4. Any other metabolic disease through medical documentation
5. Inadequate CMR quality

#### LVNC

The diagnosis of LVNC is based on previous studies^32,79^, as follows:

1. The presence of noncompacted and compacted LV myocardium with a two-layered appearance, with at least involvement of the LV apex
2. End-diastolic noncompaction/compaction ratio >2.3 on long-axis views and ≥3 on SAX views
3. Noncompacted mass >20% of the global LV mass
4. No pathologic (pressure/volume load, for example, hypertension) or physiologic (for example, pregnancy and vigorous physical activity) remodeling factors leading to excessive trabeculation

#### ARVC

The diagnostic standards for ARVC were based on the revised Task Force Criteria^80^ score with either two major criteria, one major and two minor criteria or four minor criteria. The major criteria include regional RV akinesia or dyskinesia or dyssynchronous RV contraction, ratio of RV end-diastolic volume to body surface area >110 ml m^−2^ (male) or >100 ml m^−2^ (female) or RV ejection fraction <40%; fibrous replacement of the RV free wall myocardium, with or without fatty replacement of tissue on endomyocardial biopsy; repolarization abnormalities and depolarization or conduction abnormalities on ECG test.

#### CAM

The diagnosis of CAM is based on endomyocardial biopsy or extracardiac biopsy specimens showing positive birefringence with Congo red staining under polarized light, and with native and enhanced CMR imaging in a pattern consistent with CAM: LV wall thickness of more than 12 mm shown by CMR without other known cause, with and without diffuse LGE^81^.

#### RCM

RCM is characterized by ventricular filling difficulties with increased stiffness of the myocardium. The restrictive cardiomyopathies are defined as restrictive ventricular physiology in the presence of normal or reduced diastolic volumes^52^^,82^, as follows:

1. Nondilated LV or RV with diastolic dysfunction
2. Bi-atrial dilation
3. Preserved ejection fraction (LVEF ≥50%)

We excluded subjects that met the following criteria:

1. With a reduced LV systolic function
2. Severe atrial fibrillation
3. Severe valvular disease, hypertension or congenital heart disease
4. Significant stenosis of coronary artery.

#### PAH

The diagnosis of PAH is based on the results of right heart catheterization examination. Patients are included in this study if they were clinically diagnosed as PAH^83^:

1. Mean pulmonary artery pressure (mPAP) ≥25 mmHg
2. Pulmonary capillary wedge pressure (PCWP) <15 mmHg
3. Pulmonary vascular resistance (PVR) >3 Wood units at rest

We excluded subjects with the following criteria:

1. Any evidence of cardiomyopathy, myocarditis, CAD, myocardial infarction, valvular disease, or constrictive pericarditis.
2. Any evidence of respiratory diseases.
3. History of cardiac surgery

#### Congenital heart disease—Ebstein’s anomaly

The diagnosis of Ebstein’s anomaly is based on apical displacement of tricuspid valve leaflets (≥8 mm m^−2^) with fibrous and muscular attachments to the underlying myocardium^31^. Patients with other concomitant malformation (for example, congenitally corrected transposition with Ebstein’s anomaly) and history of cardiac surgery were excluded.

#### Acute myocarditis

The diagnosis of acute myocarditis is based on the diagnostic criteria for clinically suspected myocarditis, as recommended by the European Society of Cardiology Working Group on Myocardial and Pericardial Diseases^84^, and is fulfilled by meeting the Lake Louise criteria^85^ or by confirmation through endomyocardial biopsy.

Patients with clinically acute myocarditis had the following: acute chest pain, signs of acute myocardial injury (electrocardiographic changes and/or elevated troponin level) and increased laboratory markers of inflammation (for example, C-reactive protein level). CAD was excluded before cardiac MRI. Patients with preexisting CVD were excluded.

#### HHD

The diagnostic criteria for HHD include (1) a history of prolonged, uncontrolled arterial hypertension and (2) concentric hypertrophy with left ventricular maximal wall thickness ≥12 mm.

We excluded patients with the following conditions:

1. Any other causes of LV hypertrophy
2. Cardiomyopathy
3. Obstructive coronary heart disease
4. Severe valvular disease
5. Inflammatory heart disease
6. Severe ventricular arrhythmia such as ventricular tachycardia or left bundle branch block
7. Poor CMR imaging quality

#### Normal controls

Healthy controls were recruited as volunteers without CVDs (including cardiomyopathy, CAD, severe arrhythmia or conduction block, valvular disease, congenital heart disease and so on) and other organic or systemic diseases on the comprehensive evaluation by patient history, clinical assessment, ECG and echocardiography.

### Reporting summary

Further information on research design is available in the Nature Portfolio Reporting Summary linked to this article.

## Online content

Any methods, additional references, Nature Portfolio reporting summaries, source data, extended data, supplementary information, acknowledgements, peer review information; details of author contributions and competing interests; and statements of data and code availability are available at 10.1038/s41591-024-02971-2.

### Supplementary information

Supplementary Information Supplementary Figs. 1–3 and Tables 1–7.

Reporting Summary

Supplementary Videos 1–11 Example CMR of the 11 types of CVDs.

## Figures and Tables

**Extended Data Table 1:**
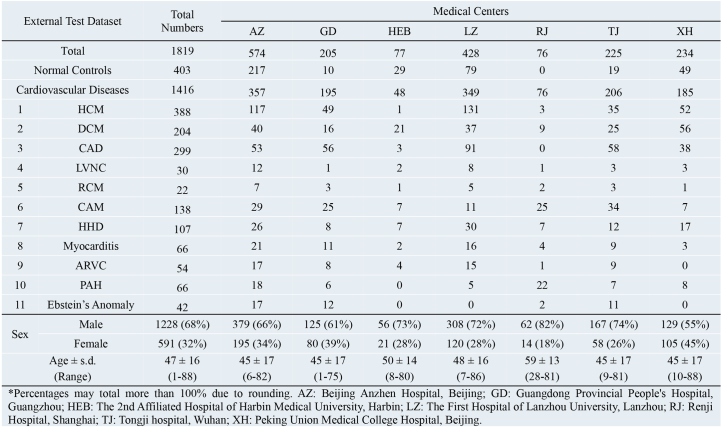
The distribution of the external validation dataset pooled from seven medical centers across China

**Extended Data Table 2:**
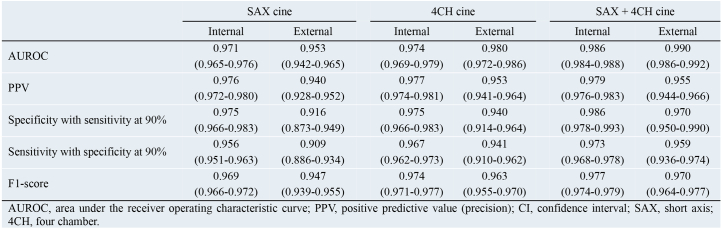
Performance summary of the screening model for anomaly detection on the primary dataset (n = 7900; three-fold cross validation) and the external dataset (n = 1819) with different CMR input schemes

**Extended Data Table 3:**
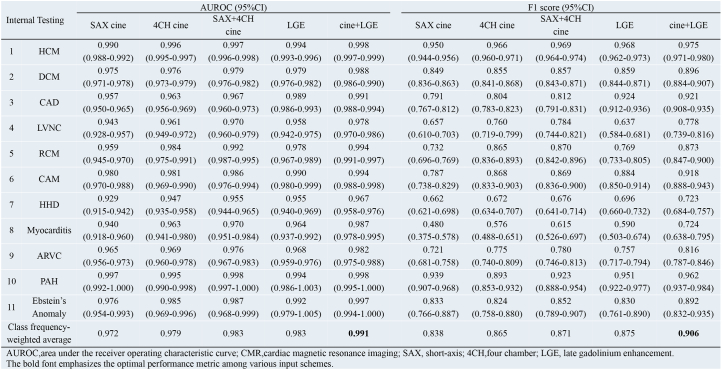
Performance of the diagnostic models with different CMR input schemes over three-fold cross validation of the primary dataset (n = 6650)

**Extended Data Table 4:**
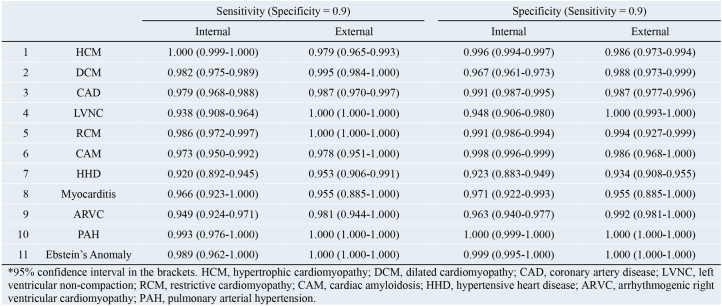
Sensitivity and specificity analysis of the diagnostic model derived from cine and LGE as combined inputs

**Extended Data Table 5:**
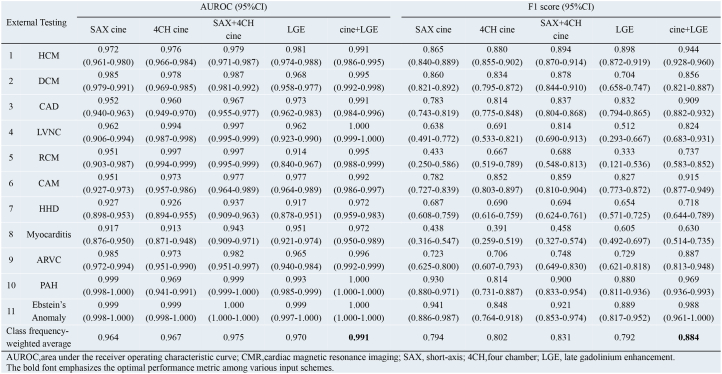
Performance of the diagnostic models with different CMR input schemes over the external test dataset (n = 1416)

**Extended Data Table 6:**
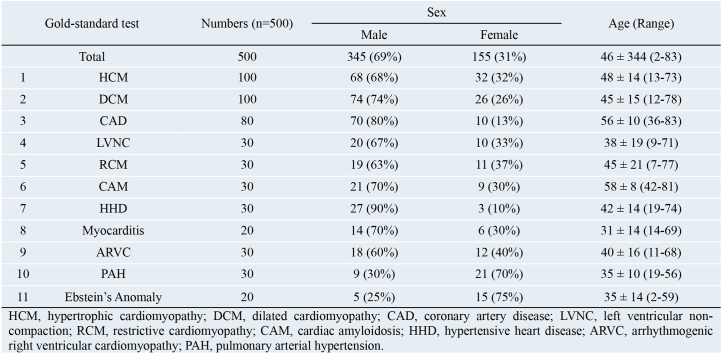
The distribution of the gold-standard test for human-AI comparison

## Data Availability

IRB approval was obtained from all participating institutions for imaging and data collection: Beijing Fuwai Hospital, China (2023–1935). The need for informed consent was waived by the respective ethics committees and institutions. No publicly available datasets were used in this study. The deidentified data can be shared only for noncommercial academic purposes and will require a formal material transfer agreement and a data use agreement. Requests should be submitted by emailing the corresponding authors (S.Z., Y.-R.J.W. or K.Z.) at cjrzhaoshihua2009@163.com, wangyanran100@gmail.com or kk.zhao@siat.ac.cn. All requests will be evaluated based on institutional policies to determine whether the data requested are subject to intellectual property or patient privacy obligations. Generally, all such requests for access to CMR data will be responded to within 1 month. Example CMR data in this study are available in supplementary videos.

## Code availability

An open-source version of the code base is available on GitHub at https://github.com/MedAI-Vision/CMR-AI with no restrictions.
