# Supplementary material for: Screening and diagnosis of cardiovascular disease using artificial intelligence-enabled cardiac magnetic resonance imaging
Source: Nat Med. 2024 May 13;30(5):1471–80. doi: 10.1038/s41591-024-02971-2 (PMC11108784; doi:10.1038/s41591-024-02971-2)

nature medicine

Article

https://doi.org/10.1038/s41591-024-02971-2

Screening and diagnosis of cardiovascular disease using artificial intelligence-enabled cardiac magnetic resonance imaging

In the format provided by the authors and unedited

Supplementary Information

Table of Contents

1. Supplementary Table 1. PPV and NPV of the diagnostic model derived from cine and LGE as combined inputs in the primary dataset (n=6650). 2

2. Supplementary Table 2. Performance of the screening model in the consecutive testing set (n=961). 2

3. Supplementary Table 3. The 48 patients from the consecutive testing set, excluded from the reported diagnostic model performance metrics. 3

4. Supplementary Table 4. Performance of the diagnostic model in the consecutive testing set (n=532). 7

5. Supplementary Table 5. Distribution of demographics and LVEF in the primary dataset. 8

6. Supplementary Table 6. Distribution of demographics and cardiac function in the consecutive testing set. 8

7. Supplementary Table 7. The typical CMR scan protocol and scanner parameters for the primary and external sets. 9

8. Supplementary Figure 1. The distribution of LVEF across the 11 CVD classes and the normal control class in the primary dataset 10

9. Supplementary Figure 2. The clinical prevalence of CVD classes 11

10. Supplementary Figure 3. The impact of learning rate modification on the VST backbone 11

1. Supplementary Table 1. PPV and NPV of the diagnostic model derived from cine and LGE as combined inputs in the primary dataset (n=6650).

Supplementary Table 1 | PPV and NPV of the diagnostic model derived from cine and LGE as combined inputs in the primary dataset (n=6650).

		PPV		NPV	
		Internal	External	Internal	External
1	HCM	0.956 (0.947-0.963)	0.932 (0.907-0.955)	0.997 (0.996-0.999)	0.983 (0.975-0.991)
2	DCM	0.875 (0.858-0.892)	0.754 (0.702-0.803)	0.977 (0.973-0.981)	0.998 (0.996-1.000)
3	CAD	0.940 (0.924-0.954)	0.952 (0.928-0.977)	0.984 (0.981-0.987)	0.966 (0.954-0.976)
4	LVNC	0.805 (0.757-0.848)	1.000 (1.000-1.000)	0.989 (0.986-0.991)	0.994 (0.989-0.998)
5	RCM	0.877 (0.843-0.912)	0.600 (0.433-0.767)	0.993 (0.990-0.995)	0.999 (0.998-1.000)
6	CAM	0.951 (0.921-0.979)	0.983 (0.955-1.000)	0.996 (0.995-0.998)	0.985 (0.978-0.991)
7	HHD	0.746 (0.704-0.789)	0.735 (0.644-0.823)	0.981 (0.977-0.984)	0.976 (0.967-0.983)
8	Myocarditis	0.776 (0.676-0.862)	0.810 (0.686-0.921)	0.996 (0.994-0.997)	0.977 (0.969-0.984)
9	ARVC	0.864 (0.825-0.899)	0.904 (0.816-0.977)	0.987 (0.984-0.989)	0.995 (0.991-0.999)
10	PAH	0.992 (0.974-1.000)	1.000 (1.000-1.000)	0.999 (0.998-0.999)	0.997 (0.994-0.999)
11	Ebstein's Anomaly	0.937 (0.875-0.986)	0.977 (0.918-1.000)	0.998 (0.997-0.999)	1.000 (1.000-1.000)

*95% confidence interval in the brackets. PPV: positive predictive value; NPV: negative predictive value.

2. Supplementary Table 2. Performance of the screening model in the consecutive testing set (n=961).

Supplementary Table 2 | Performance of the screening model in the consecutive testing set (n=961).

**Performance**	**Screening Model (SAX + 4CH cine)**
AUROC	0.984 (0.977-0.990)
PPV	0.971 (0.957-0.982)
Specificity with sensitivity at 90%	0.994 (0.965-1.000)
Sensitivity with specificity at 90%	0.946 (0.930-0.964)
F1-score	0.962 (0.953-0.972)

AUROC=area under the receiver operating characteristic curve; PPV=positive predictive value (precision); CI=confidence intervals; SAX=short axis; 4CH=four chamber.

3. Supplementary Table 3. The 48 patients from the consecutive testing set, excluded from the reported diagnostic model performance metrics.

Note: it's noteworthy that the AI screening model demonstrated robust performance by correctly classifying all 48 patients into the abnormal class, with a high average confidence score of 0.918. This successful classification, along with the high confidence score, highlights the screening model's robustness in handling a diverse range of cardiovascular diseases, including suspected phenocopies, such as genetic metabolic cardiomyopathy, which extend beyond the commonly recognized 11 CVD classes.

In contrast, the diagnostic model classified these cases with an average extremely low confidence score of 0.585, emphasizing the model's cautious approach when dealing with instances that deviate from the specified 11 CVD classes. Future direction includes the introduction of an additional AI deferral system that could defer cases with low confidence scores, falling below a predefined threshold, for expert human assessment. This collaborative synergy between human clinicians and AI models holds promise for further improving diagnostic accuracy, especially in scenarios beyond the commonly specified 11 CVD classes.

Supplementary Table 3 | The 48 patients from the consecutive testing set, excluded from the reported diagnostic model performance metrics.

Patient ID	Summary	AI Screening Model Prediction	AI Screening Confidence Score	AI Diagnostic Model Prediction	AI Diagnostic Confidence Score	Diagnosis by Human Experts
Average Confidence Score			0.918		0.585	
1	Post-operative imaging	Abnormal	0.771	RCM	0.469	Following cardiac transplant surgery, there is observed enlargement of the left atrium.
2	Post-operative imaging	Abnormal	1.000	HHD	0.648	Following surgical intervention for Hypertrophic Cardiomyopathy (HCM) utilizing the Morrow procedure.
3	Post-operative imaging	Abnormal	1.000	DCM	0.649	Patient with a history of congenital heart disease undergoing postoperative repair of a ventricular septal defect.
4	Post-operative imaging	Abnormal	0.999	ARVC	0.521	Following surgical intervention for Tetralogy of Fallot, there is observed right heart enlargement.
5	Post-operative imaging	Abnormal	1.000	PAH	0.773	Following surgical correction of atrial septal defect and pulmonary valve stenosis, there is secondary right ventricular enlargement.
6	Post-operative imaging	Abnormal	1.000	PAH	0.680	After balloon pulmonary valvuloplasty and modified Blalock-Taussig shunt procedures, there is secondary enlargement of the right atrium and ventricle.
7	Post-operative imaging	Abnormal	1.000	ARVC	0.513	Following corrective surgery for Tetralogy of Fallot, there is observed enlargement of the right ventricle.
8	Inadequate imaging quality	Abnormal	0.519	CAD	0.378	Suspected subendocardial enhancement in the left ventricular lateral wall. Unclear diagnosis
9	Inadequate imaging quality	Abnormal	1.000	RCM	0.425	Enlargement of the entire heart, reduced cardiac function, considering a correlation with atrial fibrillation.
10	Inadequate imaging quality	Abnormal	1.000	CAD	0.713	Left ventricular enlargement with impaired systolic function, demonstrating subendocardial enhancement and interventricular septal wall enhancement; a substantial likelihood of concurrent hypertension.
11	Borderline/mild cases	Abnormal	0.667	Myocarditis	0.610	Mild left ventricular enlargement is observed without evidence of fibrosis.

12	Borderline/mild cases	Abnormal	1.000	HHD	0.370	There is mild enlargement observed in both ventricles, accompanied by a slight thickening of the mid-segment of the interventricular septum (maximum thickness approximately 13mm).
13	Borderline/mild cases	Abnormal	1.000	HCM	0.667	Left ventricular enlargement with preserved systolic function is noted, and an association with sinus bradycardia is being considered.
14	Borderline/mild cases	Abnormal	0.824	Myocarditis	0.389	Mild left atrial and ventricular enlargement observed, with no evidence of fibrosis; consideration of a correlation with arrhythmia.
15	Borderline/mild cases	Abnormal	0.906	RCM	0.400	Enlargement of the left atrium is observed, along with a subtle presence of fatty signals in the left ventricular myocardium, suggestive of a mild pathological condition.
16	Borderline/mild cases	Abnormal	1.000	Myocarditis	0.704	Borderline/mild case
17	Borderline/mild cases	Abnormal	0.972	RCM	0.718	Bilateral atrial enlargement observed, with no other discernible abnormalities.
18	Borderline/mild cases	Abnormal	0.620	Myocarditis	0.455	Left ventricular enlargement with normal systolic function.
19	Borderline/mild cases	Abnormal	0.667	HCM	0.289	There is a mild thickening of the interventricular septum, measuring 12mm.
20	Dual condition	Abnormal	1.000	CAD	0.677	Diagnosis includes both CAD and DCM. CMR reveals patchy myocardial enhancement and fibrosis in subendocardial areas of the basal and mid-anterior segments of the left ventricle. Additionally, an enlarged left ventricular end-diastolic cavity measuring 60mm is observed.
21	Unclear diagnosis from human experts	Abnormal	1.000	CAD	0.673	Unclear Diagnosis from human experts
22	Unclear diagnosis from human experts	Abnormal	1.000	HHD	0.757	Interventricular septal thickening, left ventricular enlargement with reduced systolic function, and multifocal fibrosis in the septum and left ventricular lateral wall are noted. Genetic metabolic cardiomyopathy is under consideration, necessitating further investigation.
23	Unclear diagnosis from human experts	Abnormal	1.000	HHD	0.753	There is left ventricular enlargement accompanied by reduced systolic function, interventricular septal thickening, and widespread myocardial enhancement in the left ventricle. Genetic metabolic cardiomyopathy is under consideration, necessitating further investigation.
24	Unclear diagnosis from human experts	Abnormal	1.000	DCM	0.619	There is left ventricular enlargement with systolic function at the lower limit of normal, accompanied by a minor degree of fibrosis. Unclear diagnosis from human experts
25	Unclear diagnosis from human experts	Abnormal	1.000	DCM	0.713	Left ventricular enlargement is accompanied by a decrease in systolic function and the presence of multiple

						areas of myocardial fibrosis. Unclear diagnosis from human experts
26	Unclear diagnosis from human experts	Abnormal	1.000	CAD	0.691	There is left ventricular enlargement accompanied by a decrease in systolic function, along with extensive subendocardial enhancement. Unclear diagnosis from human experts
27	Unclear diagnosis from human experts	Abnormal	1.000	HHD	0.754	Noticeable thickening of both left and right ventricular walls, coupled with widespread abnormal enhancement of the left ventricular myocardium; genetic metabolic cardiomyopathy remains within diagnostic consideration.
28	Unclear diagnosis from human experts	Abnormal	1.000	DCM	0.741	Left ventricular enlargement is observed with diminished systolic function and widespread fibrosis within the left ventricular wall. Unclear diagnosis from human experts.
29	Unclear diagnosis from human experts	Abnormal	0.676	Myocarditis	0.424	Observation of left ventricular enlargement is noted, with preserved systolic function; however, a minor degree of fibrosis in the lateral wall is observed, which does not align with the diagnostic criteria for dilated cardiomyopathy (DCM).
30	Beyond the 11 CVD Classes	Abnormal	1.000	RCM	0.471	Mild left atrial enlargement is noted, accompanied by a mildly thickened left ventricular wall with multifocal fibrotic changes. This presentation is indicative of a cardiomyopathy related to a DES gene mutation.
31	Beyond the 11 CVD Classes	Abnormal	1.000	CHD	0.424	Presence of an occupying lesion in the right ventricular cavity, indicative of a tumor-like pathology, representing a rare condition.
32	Beyond the 11 CVD Classes	Abnormal	1.000	Myocarditis	0.393	Takotsubo syndrome / Stress cardiomyopathy.
33	Beyond the 11 CVD Classes	Abnormal	1.000	DCM	0.564	Constrictive pericarditis, with enlargement of both atria.
34	Beyond the 11 CVD Classes	Abnormal	0.668	HCM	0.705	The mid-segment of the interventricular septum exhibits slight thickening, while maintaining normal left ventricular systolic function. Additionally, there is a suspicion of minor subendocardial fibrosis in the left ventricular inferior wall.
35	Beyond the 11 CVD Classes	Abnormal	0.998	DCM	0.774	Mild left ventricular enlargement is observed alongside normal but reduced systolic function, indicating asynchronous left ventricular contraction. The possibility of an association with left bundle branch block (LBBB) is under consideration.
36	Beyond the 11 CVD Classes	Abnormal	1.000	HHD	0.646	Aortic valve stenosis accompanied by regurgitation, leading to secondary left ventricular enlargement and interventricular septal thickening.
37	Beyond the 11 CVD Classes	Abnormal	1.000	CAD	0.626	Bicuspid aortic valve malformation leading to secondary left ventricular enlargement, interventricular septal

						thickening, and subendocardial enhancement.
38	Beyond the 11 CVD Classes	Abnormal	0.665	DCM	0.466	Mild left ventricular enlargement is noted, with systolic function at the lower limit of normal; the presentation does not align with dilated cardiomyopathy (DCM).
39	Beyond the 11 CVD Classes	Abnormal	0.869	LVNC	0.594	Mitral valve prolapse is noted, along with left ventricular enlargement and excessive trabeculation.
40	Beyond the 11 CVD Classes	Abnormal	0.523	RCM	0.419	Bilateral atrial enlargement is noted, with consideration given to its association with atrial fibrillation.
41	Beyond the 11 CVD Classes	Abnormal	1.000	DCM	0.556	Mitral valve prolapse associated with severe regurgitation, concurrent with left ventricular enlargement.
42	Beyond the 11 CVD Classes	Abnormal	1.000	RCM	0.646	There is marked enlargement of both atria, prompting consideration of an association with atrial fibrillation.
43	Beyond the 11 CVD Classes	Abnormal	1.000	DCM	0.577	Severe aortic valve regurgitation, resulting in marked secondary enlargement of the left ventricle accompanied by reduced systolic function.
44	Beyond the 11 CVD Classes	Abnormal	1.000	DCM	0.481	Significant intrapericardial mass occupying a considerable volume within the pericardial cavity.
45	Beyond the 11 CVD Classes	Abnormal	1.000	DCM	0.618	Left ventricular enlargement with preserved function; consideration given to its association with arrhythmia (frequent premature beats).
46	Beyond the 11 CVD Classes	Abnormal	0.709	RCM	0.728	Evidence of pericardial thickening is noted, accompanied by bilateral atrial enlargement, raising suspicion for constrictive pericarditis.
47	Beyond the 11 CVD Classes	Abnormal	1.000	HHD	0.587	A quadricuspid aortic valve is observed, leading to secondary left ventricular enlargement and hypertrophy.
48	Beyond the 11 CVD Classes	Abnormal	1.000	DCM	0.634	Severe aortic valve regurgitation has led to secondary left ventricular enlargement; however, systolic function remains within an acceptable range.

4. Supplementary Table 4. Performance of the diagnostic model in the consecutive testing set (n=532).

Supplementary Table 4 | Performance of the diagnostic model in the fresh consecutive testing set (n=532).

CVD class		No. of Subjects	Diagnostic Model (cine + LGE)	
			AUROC (95%CI)	F1 score (95%CI)
1	HCM	239	0.993 (0.988-0.997)	0.958 (0.940-0.975)
2	DCM	107	0.991 (0.983-0.996)	0.922 (0.883-0.958)
3	CAD	58	0.997 (0.994-0.999)	0.915 (0.855-0.966)
4	LVNC	10	0.992	0.727
5	RCM	8	0.997	0.762
6	CAM	10	1.000	0.947
7	HHD	72	0.942 (0.904-0.970)	0.742 (0.656-1.000)
8	Myocarditis	10	0.991	0.706
9	ARVC	15	0.993	0.889
10	PAH	0	-	-
11	Ebstein's Anomaly	3	1.000	1.000
Class frequency-weighted average			0.986	0.903

AUROC=area under the receiver operating characteristic curve; CI=confidence intervals. The calculation of the 95% CI was not performed for sample sizes below 50 due to potential limitations in the precision of estimates associated with small sample sizes.

5. Supplementary Table 5. Distribution of demographics and LVEF in the primary dataset.

Supplementary Table 5 | Distribution of demographics and LVEF across 11 CVD classes and the normal control class in the primary dataset.

		No. of Subjects	Sex		Age (Range)	LVEF	
			Male	Female		Mean (STD)	Median (Q1, Q3)
	Normal Controls	1250	700 (56%)	550 (44%)	37 ± 14 (10-78)	60.1 (5.9)	60.0 (56.0, 64.0)
1	HCM	2327	1513 (65%)	814 (35%)	48 ± 14 (7-86)	65.2 (5.8)	66.0 (62.0, 69.0)
2	DCM	1435	1076 (75%)	359 (25%)	44 ± 15 (4-82)	25.9 (9.1)	25.0 (19.0, 32.0)
3	CAD	942	829 (88%)	113 (12%)	56 ± 11 (8-83)	34.8 (16.2)	33.0 (24.0, 43.0)
4	LVNC	291	192 (66%)	99 (34%)	39 ± 16 (6-77)	38.1 (14.8)	36.0 (25.9, 52.0)
5	RCM	355	170 (48%)	185 (52%)	50 ± 20 (7-85)	53.6 (8.6)	53.0 (48.0, 60.0)
6	CAM	220	156 (71%)	64 (29%)	56 ± 11 (18-83)	45.7 (11.4)	47.0 (38.1, 54.0)
7	HHD	402	366 (91%)	36 (9%)	42 ± 13 (12-75)	41.9 (15.2)	40.9 (30.1, 54.0)
8	Myocarditis	87	64 (74%)	23 (26%)	28 ± 11 (14-69)	55.3 (10.5)	57.0 (53.5, 61.0)
9	ARVC	370	245 (66%)	125 (34%)	39 ± 14 (9-74)	45.8 (13.9)	48.0 (36.0, 56.7)
10	PAH	134	36 (27%)	98 (73%)	32 ± 12 (10-72)	56.3 (7.2)	56.0 (51.9, 60.1)
11	Ebstein's Anomaly	87	33 (38%)	54 (62%)	34 ± 16 (2-63)	53.1 (9.9)	54.0 (47.8, 60.0)

*Q1: the first quartile; Q3: the third quartile; STD: standard deviation; LVEF: left ventricular ejection fraction.

6. Supplementary Table 6. Distribution of demographics and cardiac function in the consecutive testing set.

Supplementary Table 6 | Distribution of demographics and cardiac function across 11 cardiovascular disease classes and the normal control class in the independent consecutive testing set.

		Number	Sex		Age (Range)	LVEF		LV mass		LVMi		EDV		EDVi	
			Male	Female		Mean (STD)	Median (Q1, Q3)	Mean (STD)	Median (Q1, Q3)	Mean (STD)	Median (Q1, Q3)	Mean (STD)	Median (Q1, Q3)	Mean (STD)	Median (Q1, Q3)
	Total	691	465(67%)	226(33%)	45 ± 16 (2-86)	53.5(16.3)	60.0 (41.3, 66.0)	126.8(58.6)	114.0 (85.9, 161.0)	68.1(30.6)	61.1 (46.2, 83.2)	187.3(91.9)	160.0 (126.3, 219.7)	100.9(47.4)	86.0 (71.6, 115.5)
	Normal Controls	159	83(52%)	76(48%)	37 ± 16 (11-77)	63.0(5.3)	63.0 (59.7, 66.3)	77.5(25.6)	72.4 (57.6, 94.7)	42.8(11.2)	41.7 (34.3, 50.1)	138.2(33.0)	133.0 (112.3, 158.6)	76.3(13.3)	74.6 (67.4, 84.6)
1	HCM	239	160(67%)	79(33%)	49 ± 15 (7-86)	65.2(7.1)	66.0 (62.0, 70.0)	150.1(62.5)	138.8 (102.9, 179.3)	82.2(32.4)	75.8 (58.8, 100.5)	144.9(40.1)	141.0 (118.7, 164.5)	79.5(20.1)	78.0 (68.6, 89.1)
2	DCM	107	74(69%)	33(31%)	45 ± 15 (2-77)	31.3(10.1)	31.0 (22.9, 40.0)	129.9(46.5)	119.9 (96.3, 158.2)	69.3(22.5)	66.8 (53.8, 81.4)	300.4(113.2)	280.0 (216.9, 363.9)	161.8(62.0)	148.0 (121.0, 191.8)
3	CAD	58	51(88%)	7(12%)	53 ± 12 (29-81)	35.7(13.2)	33.0 (26.5, 44.5)	129.9(44.3)	121.0 (97.5, 155.0)	68.0(21.4)	62.9 (51.5, 81.8)	248.7(83.8)	231.4 (190.9, 312.1)	131.0(43.1)	123.3 (100.5, 162.2)
4	LVNC	10	7(70%)	3(30%)	35 ± 13 (17-57)	45.3(12.6)	47.5 (42.3, 55.5)	104.7(42.7)	100.2 (71.8, 123.3)	57.4(22.5)	54.9 (39.8, 66.1)	219.8(90.3)	181.2 (160.2, 282.0)	120.7(47.6)	102.3 (89.4, 144.3)
5	RCM	8	1(12%)	7(88%)	45 ± 18 (13-69)	56.5(10.3)	56.2 (53.0, 61.4)	58.4(19.3)	57.4 (47.8, 74.7)	38.2(12.6)	38.0 (31.8, 50.0)	99.1(38.9)	95.4 (75.8, 105.7)	64.9(28.6)	57.9 (50.7, 70.8)
6	CAM	10	6(60%)	4(40%)	62 ± 10 (40-73)	49.9(11.2)	49.1 (42.5, 59.5)	134.1(38.3)	124.5 (112.8, 171.5)	88.2(37.1)	75.5 (66.9, 99.5)	118.6(35.4)	121.4 (89.7, 145.6)	74.6(18.1)	82.8 (68.0, 85.3)
7	HHD	72	64(89%)	8(11%)	43 ± 13 (16-71)	44.6(13.7)	42.5 (33.9, 54.3)	168.1(60.5)	158.5 (125.3, 203.2)	84.4(34.0)	77.3 (60.0, 100.1)	236.2(93.5)	225.6 (175.5, 263.4)	117.4(48.5)	108.6 (86.5, 138.3)
8	Myocarditis	10	7(70%)	3(30%)	40 ± 19 (14-70)	54.1(11.7)	56.5 (46.0, 63.4)	99.8(31.1)	91.0 (86.0, 113.4)	54.1(16.7)	53.3 (39.8, 63.3)	160.2(39.0)	157.7 (128.3, 186.2)	84.8(18.7)	87.6 (74.4, 98.9)
9	ARVC	15	10(67%)	5(33%)	52 ± 13 (27-67)	42.3(12.4)	44.7 (35.9, 48.2)	89.6(29.0)	87.2 (64.9, 115.7)	49.2(13.4)	47.6 (37.4, 56.9)	204.6(66.0)	220.3 (162.3, 232.9)	113.3(33.5)	116.6 (88.4, 123.6)
10	PAH	0	-	-	-	-	-	-	-	-	-	-	-	-	-
11	Ebstein's Anomaly	3	2(67%)	1(33%)	33 ± 8 (25-41)	61.1(6.6)	63.6 (58.6, 64.8)	72.6(15.9)	80.7 (67.4, 81.7)	41.7(6.7)	43.6 (39.0, 45.4)	125.0(19.3)	134.7 (118.7, 136.1)	72.8(13.4)	74.3 (66.5, 79.8)

*Q1: the first quartile; Q3: the third quartile; STD: standard deviation; LV: left ventricular mass; LVMi: left ventricular mass index; EDV: end-diastolic volume; EDVi: end-diastolic volume index; LVEF: left ventricular ejection fraction.

7. Supplementary Table 7. The typical CMR scan protocol and scanner parameters for the primary and external sets.

Supplementary Table 7 | The typical CMR scan protocol and scanner parameters for the primary and external sets.

		FW			AZ	GD	HEB	LZ	RJ	TJ	XH
Manufacture		SIEMENS	GE Healthcare	Philips	Philips	Philips	Philips	Philips	Philips	SIEMENS	SIEMENS
Magnetic field strength		3	3	3	3	3	3	3	3	3	3
	Slice thickness (mm)	8	8	8	8	8	8	8	6	8	8
	Slice spacing (mm)	10	8	10	8	10	10	8	6	10	10
	Typical field of view (cm)	35	35	35	27	24	30	35	30	36	35
CINE	Echo time (ms)	1.47	1.69	1.48	1.60	1.50	1.50	1.60	1.60	1.42	1.41
	Temporal resolution (ms)	43.42	53.28	47.4	49.00	44.00	67.00	49.00	80.00	37.68	45.08
	Flip angle (degrees)	52	50	45	45	45	45	45	45	46	50
	Pixel Bandwidth (Hz/pixel)	990	488	1701	2164	1420	2188	1938	1827	965	960
	Slice thickness (mm)	8	8	8	8	8	8	8	10	8	8
	Slice spacing (mm)	9.6	8	9	8	10	10	8	10	10	10
	Typical field of view (cm)	38	35	36	27	25	30	35	30	34	35
LGE	Echo time (ms)	1.96	2.78	3	3.00	3.00	3.00	3.00	3.00	1.20	2.00
	Repetition time (ms)	6	5.98	6	6.06	6.13	6.10	6.10	6.10	6	6
	Inversion Time (ms)	300	300	300	300	300	300	350	375	280	360
	Flip angle (degrees)	20	25	25	25	25	25	25	25	55	20
	Pixel Bandwidth (Hz/pixel)	285	244	250	226	257	258	253	253	770	285

FW: Beijing Fuwai Hospital, Beijing; AZ: Beijing Anzhen Hospital, Beijing; GD: Guangdong Provincial People's Hospital, Guangzhou; HEB: The 2nd Affiliated Hospital of Harbin Medical University, Harbin; LZ: The First Hospital of Lanzhou University, Lanzhou; RJ: Renji Hospital, Shanghai; TJ: Tongji hospital, Wuhan; XH: Peking Union Medical College Hospital, Beijing.

8. Supplementary Figure 1. The distribution of LVEF across the 11 CVD classes and the normal control class in the primary dataset

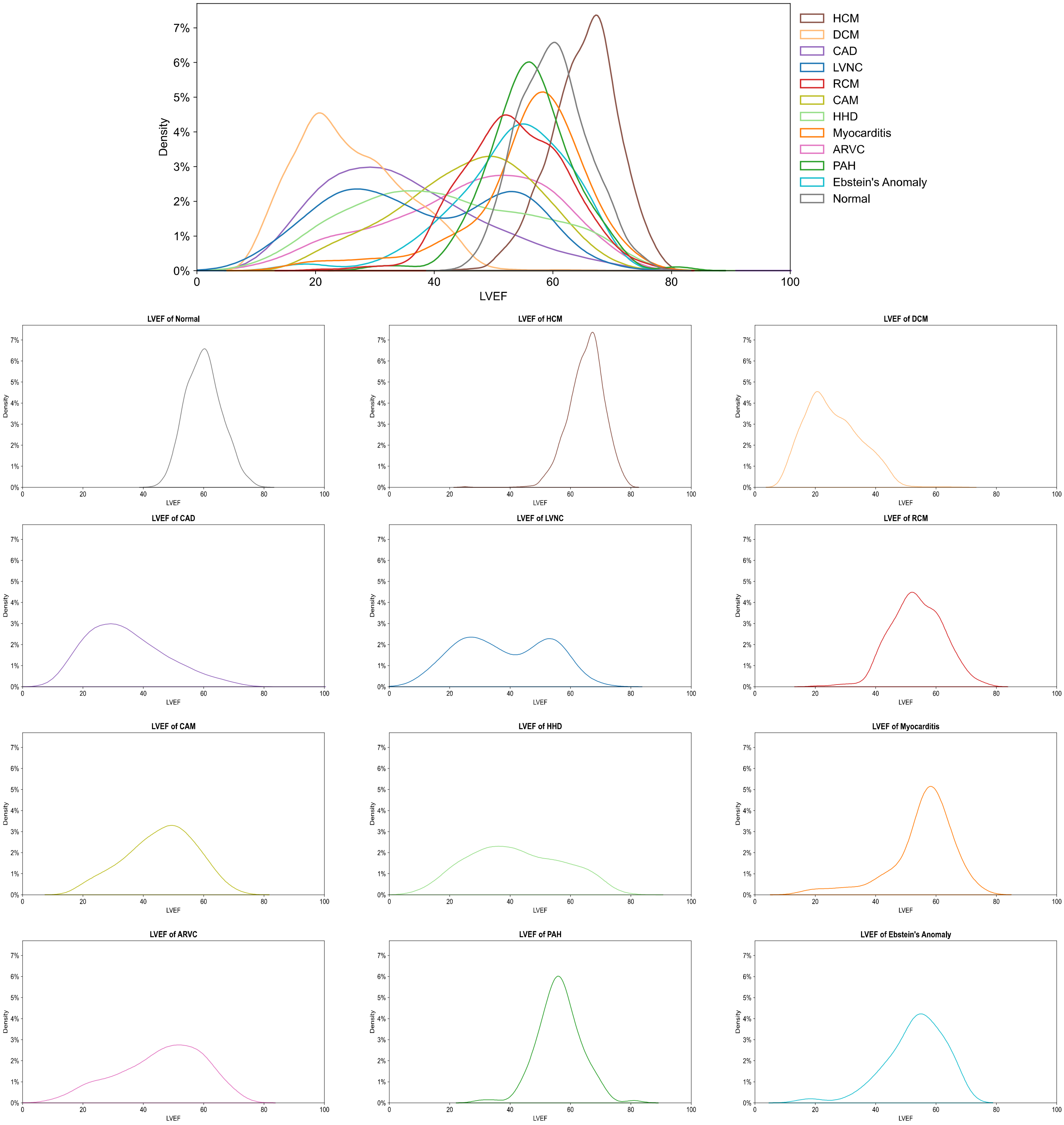

9. Supplementary Figure 2. The clinical prevalence of CVD classes

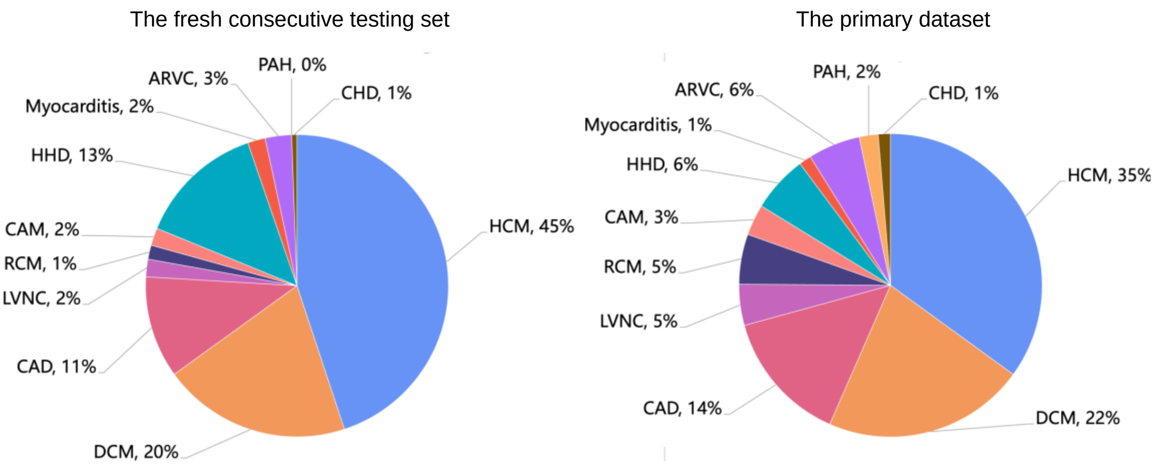

10. Supplementary Figure 3. The impact of learning rate modification on the VST backbone

The effect of modifying the initialized learning-rate (testing in one-fold of the primary cohort with the diagnostic model derived from SAX cine)

	AUROC				F1 score			
Initialized learning rate	1e-3	1e-4	1e-5	1e-6	1e-3	1e-4	1e-5	1e-6
HCM	0.992	0.989	0.990	0.987	0.941	0.945	0.937	0.914
DCM	0.973	0.975	0.972	0.962	0.825	0.849	0.817	0.788
CAD	0.959	0.962	0.949	0.901	0.747	0.757	0.728	0.589
LVNC	0.961	0.942	0.971	0.939	0.640	0.690	0.660	0.494
RCM	0.955	0.977	0.977	0.941	0.701	0.767	0.723	0.492
CAM	0.975	0.970	0.988	0.975	0.771	0.823	0.750	0.633
HHD	0.942	0.913	0.931	0.906	0.632	0.595	0.631	0.489
Myocarditis	0.936	0.967	0.980	0.943	0.367	0.490	0.510	0.432
ARVC	0.966	0.986	0.974	0.942	0.692	0.778	0.733	0.597
PAH	0.986	0.994	0.999	0.996	0.932	0.944	0.956	0.850
Ebstein's Anomaly	0.990	0.986	0.960	0.969	0.698	0.742	0.814	0.657
Class frequency-weighted	0.974	**0.974**	0.974	0.956	0.813	**0.834**	0.815	0.736

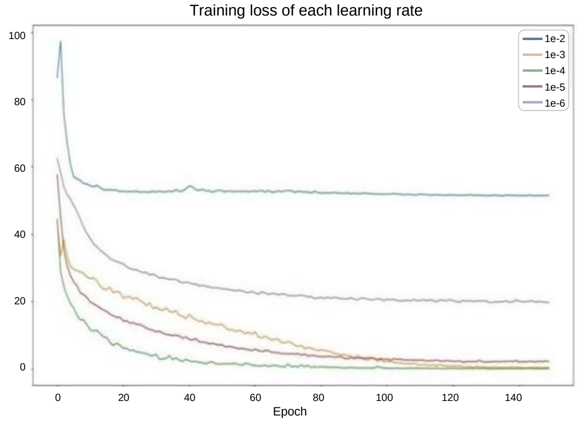

Supplement: Supplementary file 1 — Supplementary Figs. 1–3 and Tables 1–7. [file 41591_2024_2971_MOESM1_ESM.pdf]
