# Supplementary material for: Screening and diagnosis of cardiovascular disease using artificial intelligence-enabled cardiac magnetic resonance imaging
Source: Nat Med. 2024 May 13;30(5):1471–80. doi: 10.1038/s41591-024-02971-2 (PMC11108784; doi:10.1038/s41591-024-02971-2)
Supplement: Supplementary file 2 — Reporting Summary [file 41591_2024_2971_MOESM2_ESM.pdf]

nature portfolio

Corresponding author(s): Yan-Ran Joyce Wang, Ph.D.

Last updated by author(s): March 24, 2024

Reporting Summary

Nature Portfolio wishes to improve the reproducibility of the work that we publish. This form provides structure for consistency and transparency in reporting. For further information on Nature Portfolio policies, see our Editorial Policies and the Editorial Policy Checklist.

Please do not complete any field with "not applicable" or n/a. Refer to the help text for what text to use if an item is not relevant to your study.
For final submission: please carefully check your responses for accuracy; you will not be able to make changes later.

Statistics

For all statistical analyses, confirm that the following items are present in the figure legend, table legend, main text, or Methods section.

n/a	Confirmed	
☐	☒	The exact sample size (n) for each experimental group/condition, given as a discrete number and unit of measurement
☐	☒	A statement on whether measurements were taken from distinct samples or whether the same sample was measured repeatedly
☒	☐	The statistical test(s) used AND whether they are one- or two-sided<br>*Only common tests should be described solely by name; describe more complex techniques in the Methods section.*
☒	☐	A description of all covariates tested
☒	☐	A description of any assumptions or corrections, such as tests of normality and adjustment for multiple comparisons
☐	☒	A full description of the statistical parameters including central tendency (e.g. means) or other basic estimates (e.g. regression coefficient) AND variation (e.g. standard deviation) or associated estimates of uncertainty (e.g. confidence intervals)
☒	☐	For null hypothesis testing, the test statistic (e.g. F, t, r) with confidence intervals, effect sizes, degrees of freedom and P value noted<br>*Give P values as exact values whenever suitable.*
☒	☐	For Bayesian analysis, information on the choice of priors and Markov chain Monte Carlo settings
☒	☐	For hierarchical and complex designs, identification of the appropriate level for tests and full reporting of outcomes
☒	☐	Estimates of effect sizes (e.g. Cohen's d, Pearson's r), indicating how they were calculated

Our web collection on statistics for biologists contains articles on many of the points above.

Software and code

Policy information about availability of computer code

Data collection	Cardiac magnetic resonance imaging (CMR) data was collected for this study. Snap-ITK (version 4.0.1) and 3Dslicer (version 4.11.20210226) were used to visualize the CMR data and annotate the cardiac region of interests (ROI).
Data analysis	Our codes are available at https://github.com/MedAI-Vision/CMR-AI. We used Python 3.8 and PyTorch 1.10. A comprehensive list of dependencies and their version number is available at https://github.com/MedAI-Vision/CMR-AI/blob/main/requirements.txt.

For manuscripts utilizing custom algorithms or software that are central to the research but not yet described in published literature, software must be made available to editors and reviewers. We strongly encourage code deposition in a community repository (e.g. GitHub). See the Nature Portfolio guidelines for submitting code & software for further information.

Data

Policy information about availability of data

All manuscripts must include a data availability statement. This statement should provide the following information, where applicable:

- Accession codes, unique identifiers, or web links for publicly available datasets
- A description of any restrictions on data availability
- For clinical datasets or third party data, please ensure that the statement adheres to our policy

No publicly available datasets were used in this study. The de-identified data can be shared only for non-commercial academic purposes and will require a formal material transfer agreement and a data use agreement. Requests should be submitted by emailing the corresponding authors (S.Z. or Y.J.W.) at cjrzhaoshihua2009@163.com or wangyanran100@gmail.com. All requests will be evaluated based on institutional policies to determine whether the data requested are subject to intellectual property or patient privacy obligations. Generally, all such requests for access to CMR data will be responded to within 1month. Example CMR data in this study is available in Extended Data Movies.

Research involving human participants, their data, or biological material

Policy information about studies with human participants or human data. See also policy information about sex, gender (identity/presentation), and sexual orientation and race, ethnicity and racism.

Reporting on sex and gender	All reported findings apply to patients of any sex or gender.
Reporting on race, ethnicity, or other socially relevant groupings	The findings of this study are applicable to individuals of all races and ethnicities, as race, ethnicity, or other socially relevant groupings were not inclusion or exclusion criteria. However, it is important to note that all participating institutions are located in eastern Asia. Future investigations should explore the generalizability of the model across diverse ethnicities to ensure its broad utility.
Population characteristics	Table 1 and Extended Data Table 1 detailed patient demographics.
Recruitment	The study included retrospective investigation of imaging studies from patients admitted during standard clinical care. Patients were not directly recruited in this study.
Ethics oversight	The CMR datasets were acquired retrospectively under the approval of the institutional review boards (IRBs) at each participating institution, including Beijing Fuwai Hospital (FW), Beijing Anzhen Hospital (AZ), Guangdong Provincial People's Hospital (GD), the 2nd Affiliated Hospita of Harbin Medical University (HEB), the First Hospital of Lanzhou University (LZ), Renji Hospital (RJ), Tongji Hospital (TJ), and Peking Union Medical College Hospital (XH). Informed consent was waived by the IRBs. Before model training, testing, and reader studies, all data underwent de-identification processes..

Note that full information on the approval of the study protocol must also be provided in the manuscript.

Field-specific reporting

Please select the one below that is the best fit for your research. If you are not sure, read the appropriate sections before making your selection.

☒ Life sciences ☐ Behavioural & social sciences ☐ Ecological, evolutionary & environmental sciences

For a reference copy of the document with all sections, see nature.com/documents/nr-reporting-summary-flat.pdf

Life sciences study design

All studies must disclose on these points even when the disclosure is negative.

Sample size	Sample size was determined by the availability of high quality CMR and clinical data. No additional statistical method for sample size estimation was used.
Data exclusions	Data inclusion and exclusion criteria was descripted in Methods and Extended Data Figure 1. Exclusion criteria were (1) incomplete cine or LGE modalities; (2) SAX cine with fewer than 5 views; (3) CMR images with insufficient scan quality; (4) CVD patients missing clinical data; (5) CMR exams that could not be interpreted and agreed upon by the committee cardiologists according to the diagnostic criteria (Methods).
Replication	Three-fold cross validation was performed within the primary discovery cohort to further validate the model performance. We also included true external validation. We were able to replicate model performance results both internally and externally, covering eight medical centers. Hyperparameters were described in detail in Methods section for replication of model results. Confidence intervals when applicable represent variation of results or performance.
Randomization	For each three-fold cross validation, patients were randomly assigned to either the training or the validation set. For the annotation procedure, every CMR record was randomly assigned to be reviewed by a single physician (Method: Annotation procedures). For the generation of Table 2 (human-machine comparison), the 500 subjects with CMR data were randomly selected from the primary discovery cohort with a fixed CVD class ratio.
Blinding	During dataset collection, CMR experts meticulously reviewed all records and clinical reports to annotate the data with cardiovascular disease (CVD) labels, serving as ground truth for model training and evaluation. Consequently, they were not blinded to the clinical records during this process. However, in the human-machine comparison experiment involving six physicians, each physician was blinded to the interpretations of their peers and AI, as well as to the clinical and CMR reports of the patients.

Behavioural & social sciences study design

All studies must disclose on these points even when the disclosure is negative.

Study description	
Research sample	
Sampling strategy	
Data collection	
Timing	
Data exclusions	
Non-participation	
Randomization	

Ecological, evolutionary & environmental sciences study design

All studies must disclose on these points even when the disclosure is negative.

Study description

Research sample

Sampling strategy

Data collection

Timing and spatial scale

Data exclusions

Reproducibility

Randomization

Blinding

Did the study involve field work? ☐ Yes ☐ No

Field work, collection and transport

Field conditions

Location

Access & import/export

Disturbance

Reporting for specific materials, systems and methods

We require information from authors about some types of materials, experimental systems and methods used in many studies. Here, indicate whether each material, system or method listed is relevant to your study. If you are not sure if a list item applies to your research, read the appropriate section before selecting a response.

Materials & experimental systems

n/a	Involved in the study
☒	☐ Antibodies
☒	☐ Eukaryotic cell lines
☒	☐ Palaeontology and archaeology
☒	☐ Animals and other organisms
☒	☐ Clinical data
☒	☐ Dual use research of concern
☒	☐ Plants

Methods

n/a	Involved in the study
☒	☐ ChIP-seq
☒	☐ Flow cytometry
☒	☐ MRI-based neuroimaging

Antibodies

Antibodies used

Validation

Eukaryotic cell lines

Policy information about cell lines and Sex and Gender in Research

Cell line source(s)

Authentication

Mycoplasma contamination

Commonly misidentified lines
(See ICLAC register)

Palaeontology and Archaeology

Specimen provenance

Specimen deposition

Dating methods

☐ Tick this box to confirm that the raw and calibrated dates are available in the paper or in Supplementary Information.

Ethics oversight

Note that full information on the approval of the study protocol must also be provided in the manuscript.

Animals and other research organisms

Policy information about studies involving animals; ARRIVE guidelines recommended for reporting animal research, and Sex and Gender in Research

Laboratory animals

Wild animals

Reporting on sex

Field-collected samples

Ethics oversight

Note that full information on the approval of the study protocol must also be provided in the manuscript.

Clinical data

Policy information about clinical studies
All manuscripts should comply with the ICMJE guidelines for publication of clinical research and a completed CONSORT checklist must be included with all submissions.

Clinical trial registration

Study protocol

Data collection

Outcomes

Dual use research of concern

Policy information about dual use research of concern

Hazards

Could the accidental, deliberate or reckless misuse of agents or technologies generated in the work, or the application of information presented in the manuscript, pose a threat to:

No | Yes

- [ ] [ ] Public health
- [ ] [ ] National security
- [ ] [ ] Crops and/or livestock
- [ ] [ ] Ecosystems
- [ ] [ ] Any other significant area

Experiments of concern

Does the work involve any of these experiments of concern:

No | Yes

- [ ] [ ] Demonstrate how to render a vaccine ineffective
- [ ] [ ] Confer resistance to therapeutically useful antibiotics or antiviral agents
- [ ] [ ] Enhance the virulence of a pathogen or render a nonpathogen virulent
- [ ] [ ] Increase transmissibility of a pathogen
- [ ] [ ] Alter the host range of a pathogen
- [ ] [ ] Enable evasion of diagnostic/detection modalities
- [ ] [ ] Enable the weaponization of a biological agent or toxin
- [ ] [ ] Any other potentially harmful combination of experiments and agents

Plants

Seed stocks

Novel plant genotypes

Authentication

ChIP-seq

Data deposition

- [ ] Confirm that both raw and final processed data have been deposited in a public database such as GEO.
- [ ] Confirm that you have deposited or provided access to graph files (e.g. BED files) for the called peaks.

Data access links
May remain private before publication.

Files in database submission

Genome browser session
(e.g. UCSC)

Methodology

Replicates

Sequencing depth

Antibodies

Peak calling parameters

Data quality

Software

Flow Cytometry

Plots

Confirm that:

- [ ] The axis labels state the marker and fluorochrome used (e.g. CD4-FITC).
- [ ] The axis scales are clearly visible. Include numbers along axes only for bottom left plot of group (a 'group' is an analysis of identical markers).
- [ ] All plots are contour plots with outliers or pseudocolor plots.
- [ ] A numerical value for number of cells or percentage (with statistics) is provided.

Methodology

Sample preparation

Instrument

Software

Cell population abundance

Gating strategy

- [ ] Tick this box to confirm that a figure exemplifying the gating strategy is provided in the Supplementary Information.

Magnetic resonance imaging

Experimental design

Design type

Design specifications

Behavioral performance measures

Imaging type(s)

Field strength

Sequence & imaging parameters

Area of acquisition

Diffusion MRI [ ] Used [ ] Not used

Preprocessing

Preprocessing software

Normalization

Normalization template

Noise and artifact removal

Volume censoring

Statistical modeling & inference

Model type and settings

Effect(s) tested

Specify type of analysis: ☐ Whole brain ☐ ROI-based ☐ Both

Statistic type for inference

(See Eklund et al. 2016)

Correction

Models & analysis

n/a	Involved in the study
☐	☐ Functional and/or effective connectivity
☐	☐ Graph analysis
☐	☐ Multivariate modeling or predictive analysis

Functional and/or effective connectivity

Graph analysis

Multivariate modeling and predictive analysis

This checklist template is licensed under a Creative Commons Attribution 4.0 International License, which permits use, sharing, adaptation, distribution and reproduction in any medium or format, as long as you give appropriate credit to the original author(s) and the source, provide a link to the Creative Commons license, and indicate if changes were made. The images or other third party material in this article are included in the article's Creative Commons license, unless indicated otherwise in a credit line to the material. If material is not included in the article's Creative Commons license and your intended use is not permitted by statutory regulation or exceeds the permitted use, you will need to obtain permission directly from the copyright holder. To view a copy of this license, visit http://creativecommons.org/licenses/by/4.0/
